# Hsp70 Isoforms Are Essential for the Formation of Kaposi’s Sarcoma-Associated Herpesvirus Replication and Transcription Compartments

**DOI:** 10.1371/journal.ppat.1005274

**Published:** 2015-11-20

**Authors:** Belinda Baquero-Pérez, Adrian Whitehouse

**Affiliations:** School of Molecular and Cellular Biology and Astbury Centre for Structural Molecular Biology, University of Leeds, Leeds, United Kingdom; University of California, Los Angeles, UNITED STATES

## Abstract

Kaposi’s sarcoma-associated herpesvirus (KSHV) is an oncogenic herpesvirus associated with various AIDS-related malignancies. Like other herpesviruses, multiple processes required for KSHV lytic replication, including viral transcription, viral DNA synthesis and capsid assembly occur in virus-induced intranuclear structures, termed replication and transcription compartments (RTCs). Here we utilised a novel methodology, combining subcellular fractionation and quantitative proteomics, to identify cellular proteins which are recruited to KSHV-induced RTCs and thus play a key role in KSHV lytic replication. We show that several isoforms of the *HSP70* chaperone family, Hsc70 and iHsp70, are redistributed from the cytoplasm into the nucleus coinciding with the initial formation of KSHV-induced RTCs. We demonstrate that nuclear chaperone foci are dynamic, initially forming adjacent to newly formed KSHV RTCs, however during later time points the chaperones move within KSHV RTCs and completely co-localise with actively replicating viral DNA. The functional significance of Hsp70 isoforms recruitment into KSHV RTCs was also examined using the specific Hsp70 isoform small molecule inhibitor, VER-155008. Intriguingly, results highlight an essential role of Hsp70 isoforms in the KSHV replication cycle independent of protein stability and maturation. Notably, inhibition of Hsp70 isoforms precluded KSHV RTC formation and RNA polymerase II (RNAPII) relocalisation to the viral genome leading to the abolishment of global KSHV transcription and subsequent viral protein synthesis and DNA replication. These new findings have revealed novel mechanisms that regulate KSHV lytic replication and highlight the potential of *HSP70* inhibitors as novel antiviral agents.

## Author Summary

Molecular chaperones from the *HSP70* and *HSP90* families have important roles in cell survival. Recent evidence has also implicated their functioning in a variety of diseases, including cancer. As such they have been identified as emerging drug targets. Kaposi’s sarcoma-associated herpesvirus (KSHV) is an oncogenic herpesvirus which, like other herpesviruses, lytically replicates in virus-induced structures within the nucleus, termed replication and transcription compartments (RTCs). Here we developed a novel proteomic approach enhanced by subcellular fractionation to study the cellular protein composition of KSHV-induced RTCs. Results revealed that the constitutively expressed Hsc70 and the stress-inducible iHsp70 chaperones were significantly increased in the KSHV-induced RTCs. Importantly, inhibition of the ATPase function of these chaperones led to a marked reduction in KSHV RTCs formation and KSHV lytic replication. Notably, these results highlight the therapeutic potential of *HSP70* inhibitors for the treatment of KSHV-related diseases, such as Kaposi’s sarcoma.

## Introduction

Molecular chaperones represent a large group of proteins that are essential for maintaining cellular homeostasis and survival. As such, the roles of these proteins are numerous; facilitating correct protein folding or unfolding, assembly or disassembly of multimeric protein complexes, participating in translocation of proteins and vesicles into organelles, stabilising a wide range of signalling molecules and preventing aggregation of non-native proteins (reviewed in [1, 2]). Heat shock proteins (HSP) are classified according to their molecular weight into several families: *HSP40*, *HSP60*, *HSP70*, *HSP90*, *HSP100*, *HSP110* and the small HSP (less than 34 kDa)[3]. The functional importance of the *HSP70* and *HSP90* families of molecular chaperones is exemplified by their emerging implications in a variety of diseases, including cancer [4, 5], neurodegeneration [6] or viral infection [7, 8]. As such they have gained significant interest recently as potential drug targets.

Eukaryotes have multiple genes encoding for chaperones of the *HSP70* family, which are amongst the most conserved proteins in evolution [9–11]. The major Hsp70 isoforms are the constitutively expressed Hsc70, the stress-inducible Hsp70 (iHsp70), the endoplasmic reticulum resident (Grp78) and the mitochondrial form (Grp75). All Hsp70 isoforms have an N-terminal domain which harbours a highly conserved ATPase and a C-terminal substrate binding domain [1]. Hsp90 isoforms which comprise the inducible and constitutively-expressed isoforms (Hsp90α and Hsp90β respectively), the ER resident (Grp74) and the mitochondrial form (TRAP1), also possess a N-terminal ATP binding domain, although this has no similarity to the ATP-binding domain found in the chaperones of the *HSP70* family [5]. The presence of ATPase pockets in both families of chaperones makes these proteins desirable targets for small molecule inhibitors [12, 13]. The therapeutic potential of these compounds is especially evident for several *HSP90* inhibitors, having already reached phase II and III clinical trials [14, 15]. Targeting of Hsp70 isoforms has been more challenging [12], but recently specific inhibitors have also undergone clinical trials [16, 17]. Importantly, the development of highly specific inhibitors for Hsp70 isoforms may have potential for the treatment of a diverse group of viruses as the functional importance of Hsp70 isoforms in the life cycle of numerous viruses has been highlighted over the past few years [8]. Distinct Hsp70 isoforms are usurped to aid in many stages of viral replication as varied as viral entry, uncoating, transcription, envelope protein maturation, morphogenesis or DNA replication [8]. Therefore, the importance of these chaperones in the life cycle of such a wide range of viruses suggests the potential of these proteins as targets for broad-spectrum antivirals.

Kaposi’s sarcoma-associated herpesvirus (KSHV) is the causative agent of several AIDS-associated malignancies, including Kaposi’s sarcoma (KS), a highly vascular tumour of endothelial lymphatic origin. Similar to other herpesviruses, KSHV exists in two distinct life cycles, latent persistence or lytic replication phases. However, unlike other human oncogenic viruses where the latent cycle is predominantly responsible for tumorigenesis, both the latent and lytic replication phases are essential for KSHV tumorigenicity in KS [18, 19]. During latency, which is established in B cells and in the tumour setting, viral gene expression is highly restricted, only involving expression of the latency-associated nuclear antigen (LANA), the viral FLICE inhibitory protein, viral cyclin, kaposins and several virally-encoded miRNAs [20]. However, upon reactivation, the virus enters the lytic reactivation cycle leading to expression of more than 80 protein-coding sequences and replication of viral genomes [21] in a highly orchestrated sequential manner. Infectious virions can then spread to endothelial cells where tumours develop. Moreover in KS lesions, where most infected cells harbour the virus in a latent state, a small proportion of cells undergo lytic replication which leads to secretion of lytically-expressed angiogenic, inflammatory and proliferative factors that act in a paracrine manner on latently-infected cells enhancing tumourigenesis [20]. Furthermore, lytic replication enhances genomic instability [22] and also sustains the population of latently-infected cells that would otherwise be reduced due to the poor persistence of KSHV episomes during cell division [23]. Therefore, inhibiting KSHV lytic replication may lead to a novel therapeutic intervention for the treatment of KSHV-associated diseases.

KSHV initiates lytic replication upon transcription of the *ORF50/RTA* gene which encodes the conserved lytic master switch RTA protein. RTA is then able to trigger the entire lytic gene expression cascade in KSHV and other γ-2 herpesviruses [24, 25]. KSHV transcription, DNA replication and packaging, and capsid assembly all occur in virus-induced nuclear structures, termed replication and transcription compartments (RTCs) [26]. Early in herpesvirus infection, viral transcription of early genes and viral DNA replication takes place in small RTCs [27], that generally concentrate at the nuclear periphery [28–30]. As infection progresses, the nuclear architecture undergoes a striking re-organization to facilitate viral replication. Small RTCs coalesce into single large globular or kidney-shaped structures that ultimately fill most of the nuclear space compressing and marginalizing the cellular chromatin to the nuclear periphery [28, 29]. These large RTCs support late viral gene expression, viral DNA synthesis and capsid assembly [27]. Previous efforts to identify the protein composition, both viral and cellular, of herpesvirus RTCs have been carried out using immunoprecipitation-based assays, identifying proteins that associate either with the KSHV lytic origin of DNA replication (*ori-Lyt*) [31] or with known HSV-1 viral proteins which accumulate in RTCs [32]. However, these immunoprecipitation-based approaches restrict the number of proteins identified and are not quantitative, thus a more quantifiable and high-throughput method is highly desirable.

In recent years, the use of shotgun proteomics has proved an invaluable tool to investigate global analysis of protein composition, allowing the elucidation of new aspects of viral biology [33]. An enhanced approach to this methodology is the combined use of subcellular fractionation with shotgun proteomics [34]. This allows the identification of cellular proteins that can easily be masked by more abundant proteins in studies which interrogate the global proteome using whole cell lysates or large cellular regions (e.g. nuclear or cytoplasmic). Therefore, we have utilised a novel quantitative proteomic approach to identify cellular proteins which are either recruited to or present at significant levels in KSHV-induced RTCs and thus play a key role during KSHV lytic replication. Herein, we have utilised subcellular fractionation coupled to stable isotope labelling by amino acids in cell culture (SILAC)-based quantitative proteomics followed by liquid chromatography (LC)-tandem mass spectrometry (MS/MS). We uniquely isolated the nuclear envelope (NE) region from unreactivated (harbouring latent virus) and KSHV-reactivated (harbouring lytic virus) cells using a recently developed method [35] and notably demonstrate the enrichment of purified RTCs. A rationale of this approach is that the NE cannot be purified completely due to its multiple subcellular connections. The outer nuclear membrane is continuous with the endoplasmic reticulum and interacts with the cytoskeleton [36, 37] while the inner nuclear membrane binds to host chromatin [38–40]. Thus, we took advantage of its incomplete purity so that we could isolate not only the NE and embedded nuclear pore complexes, but also components found in the NE neighbourhood, such as RTCs. Utilising this novel approach we demonstrate that cellular chaperones from the *HSP70* family (Hsc70 and iHsp70) are significantly increased in the NE-associated RTCs of reactivated cells. Functional dissection further demonstrates that these chaperones were specifically recruited to the periphery of incipient RTCs coinciding in time with their formation. When actively replicated viral DNA was synthesised the chaperones were recruited within RTCs. Strikingly, inhibition of Hsp70 isoforms precluded RTC formation, curtailed chaperone redistribution within RTCs and RNAPII recruitment to viral promoters. Importantly, abrogation of lytic replication occurred without affecting cell viability, suggesting that the cellular housekeeping functions carried out by these chaperones were not compromised. As such, *HSP70* inhibitors may provide a novel therapeutic approach for the treatment of KSHV-associated malignancies, in particular it would be interesting to determine the efficacy of combining the potential of inhibiting lytic replication using *HSP70* inhibitors with the previous reported effect of *HSP90* inhibitors to eradicate latent KSHV reservoirs [41].

## Results

### The cellular chaperones Hsc70, iHsp70 and Grp78 were enhanced in the NE-associated RTCs of reactivated HEK-293T rKSHV.219 cells

**Fig 1:**
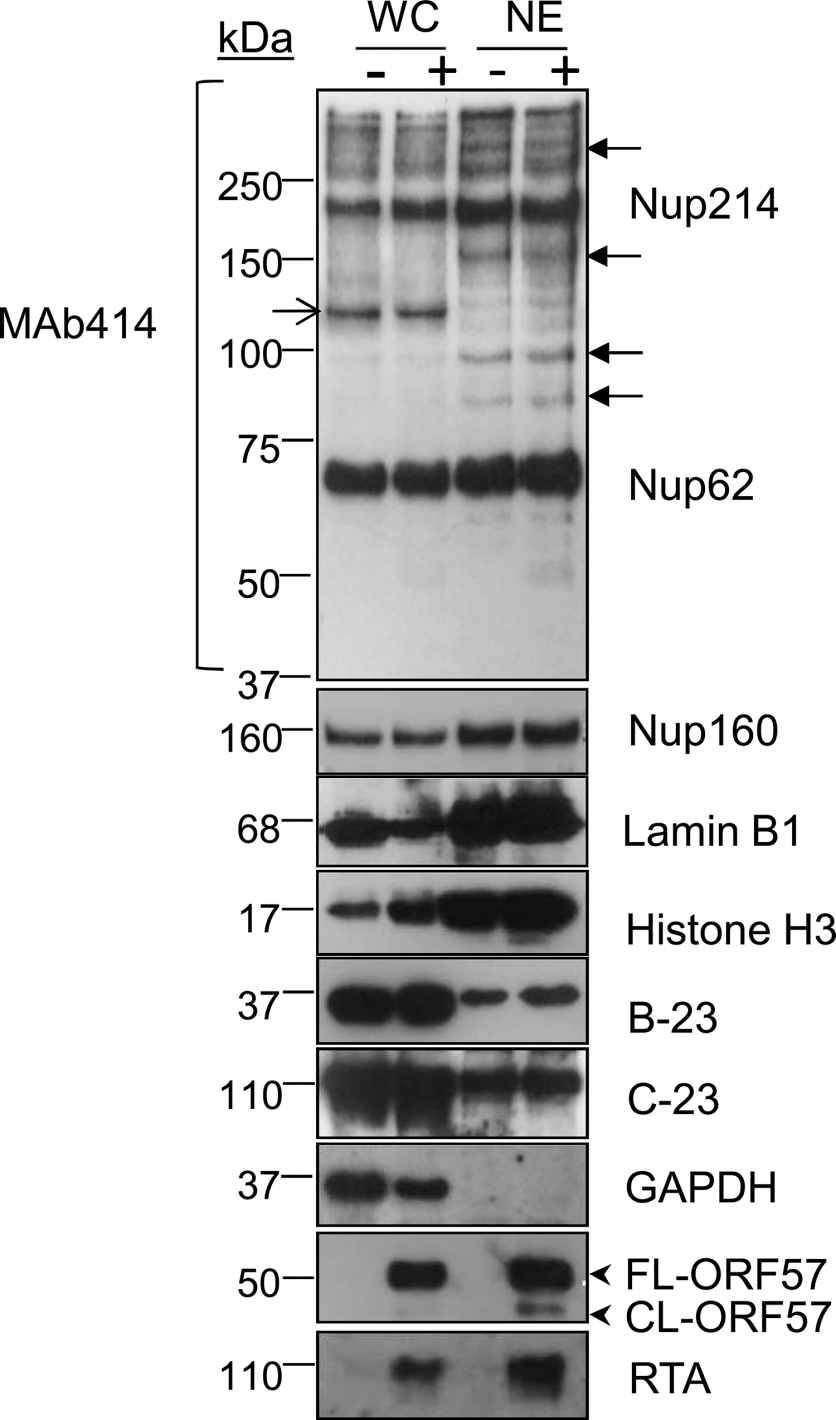
Successful enrichment of the nuclear envelope region and associated KSHV RTCs in HEK-293T rKSHV.219 cells. Successful enrichment of NE regions in unreactivated (-) and reactivated for 48 h (+) HEK-293T rKSHV.219 cells was demonstrated by Western blotting. Equal amounts of total protein extracted from whole cell (WC) and nuclear envelope (NE) fractions were used. The monoclonal antibody Mab414 specifically recognises the phenylalanine-glycine (FG)-repeat motif present in numerous nucleoporins (Nups). Closed arrows point to Nups that were only detected by MAb414 after NE enrichment. Open arrow points to a nucleoporin lost during NE enrichment. Several FG Nups, together with Nup160, histone H3 and lamin B1 were all markedly enriched in the NE fractions compared with WC fractions. The nucleolar proteins B-23 and C-23 and the cytoplasmic GAPDH protein were all decreased in NEs fractions indicating correct subcellular fractionation. The viral RTC-associated ORF57 and RTA proteins were used to monitor lytic reactivation and assess RTC enrichment. ORF57 antibody detected both full length ORF57 protein (FL-ORF57) and the caspase-7-cleaved ORF57 (CL-ORF57).

To investigate differential proteome changes which occur during KSHV lytic replication in NE-associated RTCs, we utilised the HEK-293T cell line containing a latent recombinant KSHV virus (rKSHV.219) [42]. This cell line can be reactivated into a full lytic replication cycle via chemical induction. Unreactivated cells were grown in isotopically labelled media (R6K4), while cells to be reactivated were grown in label-free media (R0K0). After isotopic labelling was complete, cells were reactivated for 48 h and nuclear envelopes (NEs) were then successfully purified using a recently described method [35], with minor modifications. Western blot analysis of the NE preparations demonstrated an enrichment of nucleoporins (Nups), lamins and histones and a loss of cytoplasmic (GAPDH) and nucleolar (B-23, C-23) proteins compared with whole cell (WC) lysates (**Fig 1**). The essential KSHV mRNA processing protein, ORF57 [43], and viral RTA served as markers for lytic viral replication and RTC enrichment, as both of these proteins are known to be recruited to KSHV RTCs [31, 44]. The monoclonal antibody (Mab414), which recognises the phenylalanine-glycine (FG)-repeat motif present in numerous Nups, and the polyclonal antibodies against Nup160 and lamin B1 were used to assess enrichment of the NE region. NE preparations showed a higher number of Nups (closed arrows) than their respective WC preparations; although some Nups were lost following 0.3 M salt wash (open arrow). Following LC-MS/MS analysis and using a minimum of three unique peptides assigned to a single protein, most proteins (1072) remained unchanged in their abundance irrespectively of KSHV lytic infection and only five proteins had a significant reduction (ratio cut-off <0.5) in NEs of reactivated cells. In contrast, 216 proteins showed a significant increase (ratio cut-off >1.9) during lytic replication. Importantly, multiple cellular proteins that are known or expected to localize within herpesvirus RTCs; such as those associated with KSHV *ori*-*Lyt*, the HCMV transactivator IE2-p86 protein or the herpes simplex virus-1 (HSV-1) ICP8 protein were found significantly increased in the NE regions of reactivated cells using the less stringent ratio cut-off of 1.5 (**S1 Table**). Some of these cellular proteins included CSNK2A1 [45], BLM [32], topoisomerases I and II [31, 46, 47] and DEAD box helicases DDX5 [32] and DDX17 [45]. Thus, LC-MS/MS results confirmed the correct isolation of the NE region and accompanying RTCs. Importantly, many of the 216 identified proteins most likely represent novel cellular proteins hijacked by KSHV, not only due to the subcellular fractionation carried out but also to the uncommon use of urea for protein extraction before LC-MS/MS. All proteins identified by LC-MS/MS can be seen on **S1 Dataset**. Bioinformatical analysis revealed several upregulated pathways (ratio cut-off >1.9) in reactivated cells (**S2 Table**). Of particular interest was an upregulated pathway which related to protein folding. This included several Hsp70 isoforms and their associated co-chaperones from the *HSP40* (DNAJ) family (**Table 1**).

**Table 1:** SILAC results from NE fractions highlighted chaperones from the *HSP70* family and their associated co-chaperones

Protein	UniProt number	Ratio Reactivated/ Unreactivated	Unique peptides
Hsp70-1/Hsp70-2 (iHsp70)	P08107	6.6	1
Hsc70	P11142	4.1	41
Grp78 (BiP)	P11021	3.64	16
DNAJA1	P31689	4.158	12
DNAJB1	P25685	21.831	7
DNAJB6	B4DVN1	5.338	5

Notably, the constitutively expressed chaperone Hsc70 presented a 4.1-fold increase with 41 unique peptides assigned. This protein had the highest fold increase associated with the most unique peptide number of all the proteins identified by LC-MS/MS. This could be due to increased Hsc70 expression or to the redistribution of Hsc70 from the cytoplasm into the NE region during KSHV lytic replication. Therefore, due to the vital importance of Hsp70 isoforms in the replication cycle of a wide range of viral families, we focussed our studies herein on the roles of the three main Hsp70 isoforms (Hsc70, iHsp70 and Grp78) during KSHV lytic replication.

### Hsc70 and iHsp70 are redistributed from the cytoplasm to both the periphery and within KSHV-induced RTCs

**Fig 2:**
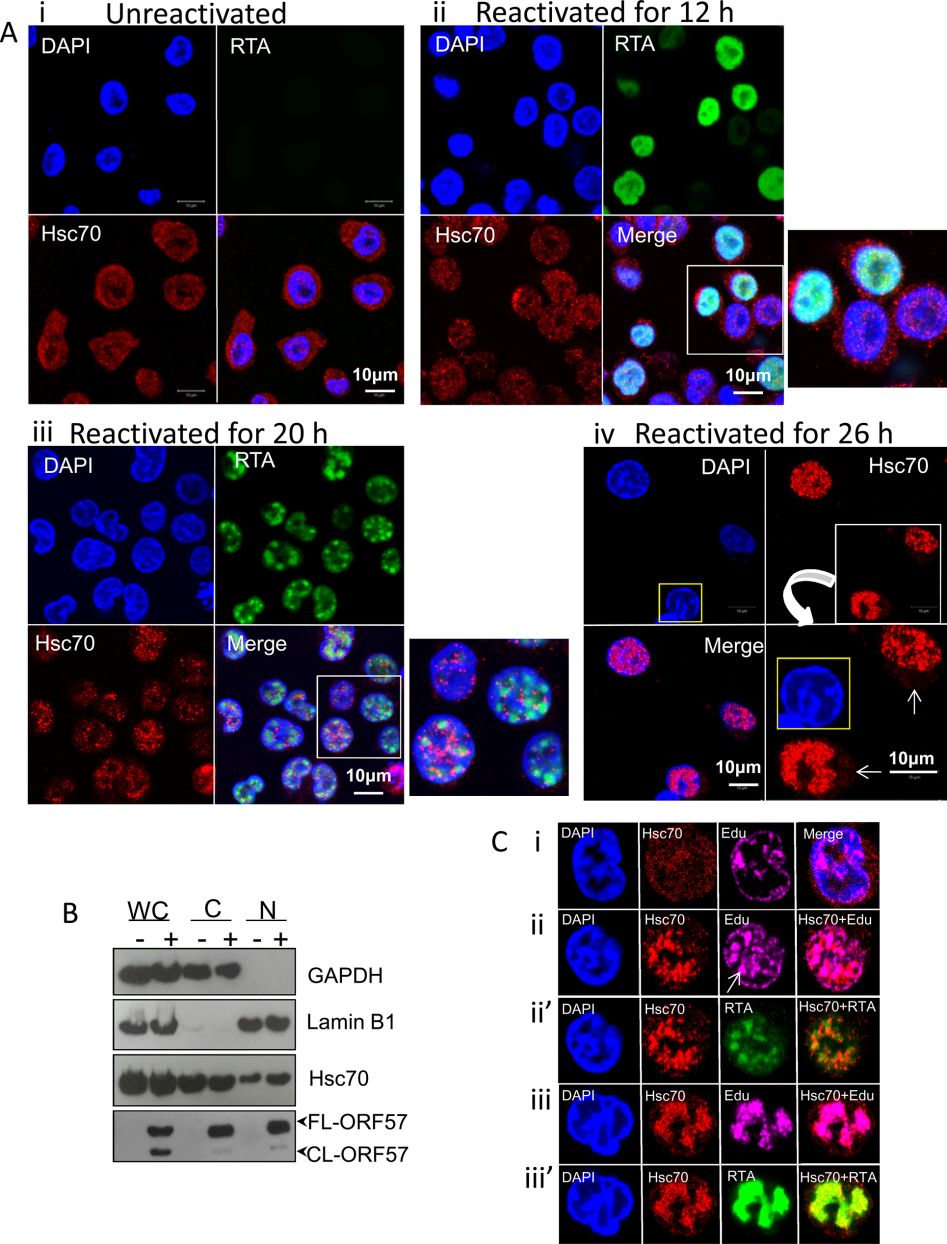
Hsc70 was redistributed from the cytoplasm to both the periphery and within KSHV-induced RTCs. (A) Unreactivated TREx BCBL1-RTA cells exhibited endogenous Hsc70 protein equally distributed between the cytoplasm and the nucleus (i). The same Hsc70 location was seen at 12 h reactivation prior to RTC formation, when RTA was diffuse in the nucleus (ii). At 20 h reactivation viral RTA was assembled into incipient RTCs. Hsc70 was not detected in the cytoplasm and instead numerous nuclear foci that were positioned predominantly adjacent to RTCs were seen (iii). At later reactivation times very large Hsc70 foci were completely recruited within RTCs. Hsc70 cytoplasmic depletion is indicated (iv arrows). Nucleus highlighted in yellow shows a typical KSHV fully developed-RTC with cellular chromatin marginalised to the nuclear periphery. (B) Unreactivated (-) or reactivated for 26 h (+) TREx BCBL1-RTA cells were fractionated into whole cell (WC), cytoplasmic (C) and nuclear (N) fractions. Equal amounts of total protein from each fraction were analysed by Western blotting. Nuclear fractions were characterised by enrichment of lamin B1 and absence of cytoplasmic GAPDH, while cytoplasmic fractions showed the inverse profile. A small decrease in cytoplasmic Hsc70 which correlated with a small increase in nuclear Hsc70 was detected in reactivated cells, further supporting that Hsc70 was redistributed from the cytoplasm to the nucleus. (C) TREx BCBL1-RTA cells remained unreactivated (i) or reactivated for 24 h (ii-iii’) followed by triple-labelling with antibodies specific for RTA and Hsc70 and Click-iT EdU Alexa Fluor 647, the latter allowed visualization of newly synthesized DNA. During early reactivation, a proportion of Hsc70 protein was adjacent to small viral RTCs. Arrow points to a small KSHV RTC filled with viral DNA (ii). Hsc70 also partially co-localised with viral DNA (ii) and RTA (ii’) in small RTCs. During later reactivation times, Hsc70 completely moved within fully-developed RTCs strongly co-localising with viral DNA (iii) and RTA (iii’).

To verify the enrichment of Hsc70 in the NE-associated RTCs of reactivated cells detected by our quantitative proteomic approach, indirect immunofluorescence was used to label endogenous Hsc70 protein in TREx BCBL1-RTA cells, a KSHV latently infected B-lymphocyte cell line containing a Myc-tagged version of viral RTA under the control of a doxycycline-inducible promoter [48]. Hsc70 protein was equally distributed between the cytoplasm and nucleus of unreactivated cells in a fine punctuate pattern (**Fig 2Ai**). Similar Hsc70 localization was seen during early lytic replication (12 h reactivation), when RTA protein was diffuse in the nucleus, prior to RTC formation (**Fig 2Aii**). In contrast, at later reactivation time points (20 h), in which RTA was organised into small viral RTCs peripherally located in the nucleus, numerous nuclear Hsc70 foci that were predominantly adjacent to RTCs were observed (**Fig 2Aiii**). During late reactivation time points (26 h), cellular chromatin was marginalised to the nuclear periphery (**Fig 2Aiv**, nucleus highlighted in yellow) and larger Hsc70 foci avidly accumulated within these fully-developed RTCs (**Fig 2Aiv**). Reduced levels of cytoplasmic Hsc70 were also observed at this time point (**Fig 2Aiv arrows**), suggesting that Hsc70 is redistributed from the cytoplasm to the nucleus during KSHV lytic replication. This is further supported by the fact that fractionation of TREx BCBL1-RTA cells into nuclear (N) and cytoplasmic (C) fractions displayed an enrichment of Hsc70 in the nuclei of reactivated cells which occurred without a noticeable increase in Hsc70 protein levels in whole cell (WC) lysates (**Fig 2B**). Due to the observed redistribution of Hsc70 to KSHV RTCs, further co-localization studies between Hsc70 and the sites of viral DNA replication were performed in TREx BCBL1-RTA cells. Here, cells were triple-labelled with Click-iT EdU Alexa Fluor 647 and antibodies specific for RTA and Hsc70. In unreactivated cells, newly synthesized cellular DNA during mid-S-phase (EdU incorporated) occurred mainly at the nuclear periphery as previously observed in other cell types [49, 50] (**Fig 2Ci**). During early reactivation, Hsc70 was adjacent to RTA which was present in small viral RTCs containing actively replicating viral DNA (**Fig 2Cii arrow**). At this stage, a proportion of Hsc70 also co-localised with RTA (**Fig 2Cii’**). During late reactivation, when cellular chromatin was marginalised, much larger RTCs were visible and newly synthesized cellular DNA was not apparent, here Hsc70 completely co-localised with newly synthesized viral DNA and RTA (**Fig 2Ciii and 2Ciii’**).

The location of the other two main Hsp70 isoforms (iHsp70 and Grp78) during KSHV lytic replication was also investigated by indirect immunofluorescence microscopy. iHsp70 was cytoplasmic in unreactivated TREx BCBL1-RTA cells (**S1Ai Fig**), in contrast, an increase in nuclear iHsp70 labelling was observed in reactivated cells, which displayed similar chaperone foci as those seen during early reactivation for Hsc70 (**S1Aii Fig**). Occasionally, cells displayed RTCs completely filled by iHsp70 (**S1Aiii and S1Aiv Fig, asterisks**). Large iHsp70 foci positioned adjacent to RTCs were also seen in reactivated cells at later time points (**S1Aiv Fig arrows**). Co-localisation of iHsp70 with actively replicating viral DNA was also observed during late reactivation (**S1B Fig**). Hsc70 and iHsp70 nuclear foci appeared at the same time as KSHV RTCs were assembled, suggesting that these chaperones could be involved in RTC assembly. Additionally, complete co-localization of Hsc70 and iHsp70 with viral DNA indicated that these chaperones could also participate in viral DNA replication and/or capsid assembly. In contrast, the endoplasmic reticulum (ER) Hsp70 isoform, Grp78, was not redistributed in reactivated TREx BCBL1-RTA cells (**S2 Fig**), consistent with its ER retention signal [51]. Nevertheless, reactivated cells seemed to accumulate larger amounts of Grp78 in the ER. To confirm these results, immunoflourescence studies were also performed using HEK-293T rKSHV.219 cells, in which the presence of the recombinant virus is tracked by expression of the green fluorescent protein (GFP) from the EF-1alpha promoter and lytic reactivation levels are monitored by expression of the red fluorescent protein (RFP) from the KSHV lytic non-coding polyadenylated nuclear (PAN) RNA promoter. Unreactivated cells displayed cytoplasmic iHsp70 and Hsc70 labelling, whereas _~_ 40% of reactivated cells (24 h reactivation) revealed nuclear iHsp70 and Hsc70 accumulations that appeared to assemble within small RTCs (**S3A Fig arrows and S3B Fig arrows** respectively). The incomplete redistribution of iHsp70 and Hsc70 foci into RTCs was likely due to a more asynchronous progression through the lytic cycle in induced cells by TPA and sodium n-butyrate than in doxycycline-induced TREx BCBL1-RTA cells. Similarly to TREx BCBL1-RTA cells, Grp78 was not redistributed in HEK-293T rKSHV.219 cells, although larger amounts appeared to accumulate in the ER of reactivated cells (**S3C Fig**), in agreement with the significantly increased amounts of Grp78 detected in the NE region of these cells (**Table 1**). These results clearly demonstrate that KSHV specifically redistributes the molecular chaperones, Hsc70 and iHsp70, from the cytoplasm to the nucleus, in contrast to Grp78, which coincides with the initial formation of KSHV RTCs.

### Treatment with the small molecule inhibitor VER-155008 abrogated viral protein synthesis at non-cytotoxic concentrations

**Fig 3:**
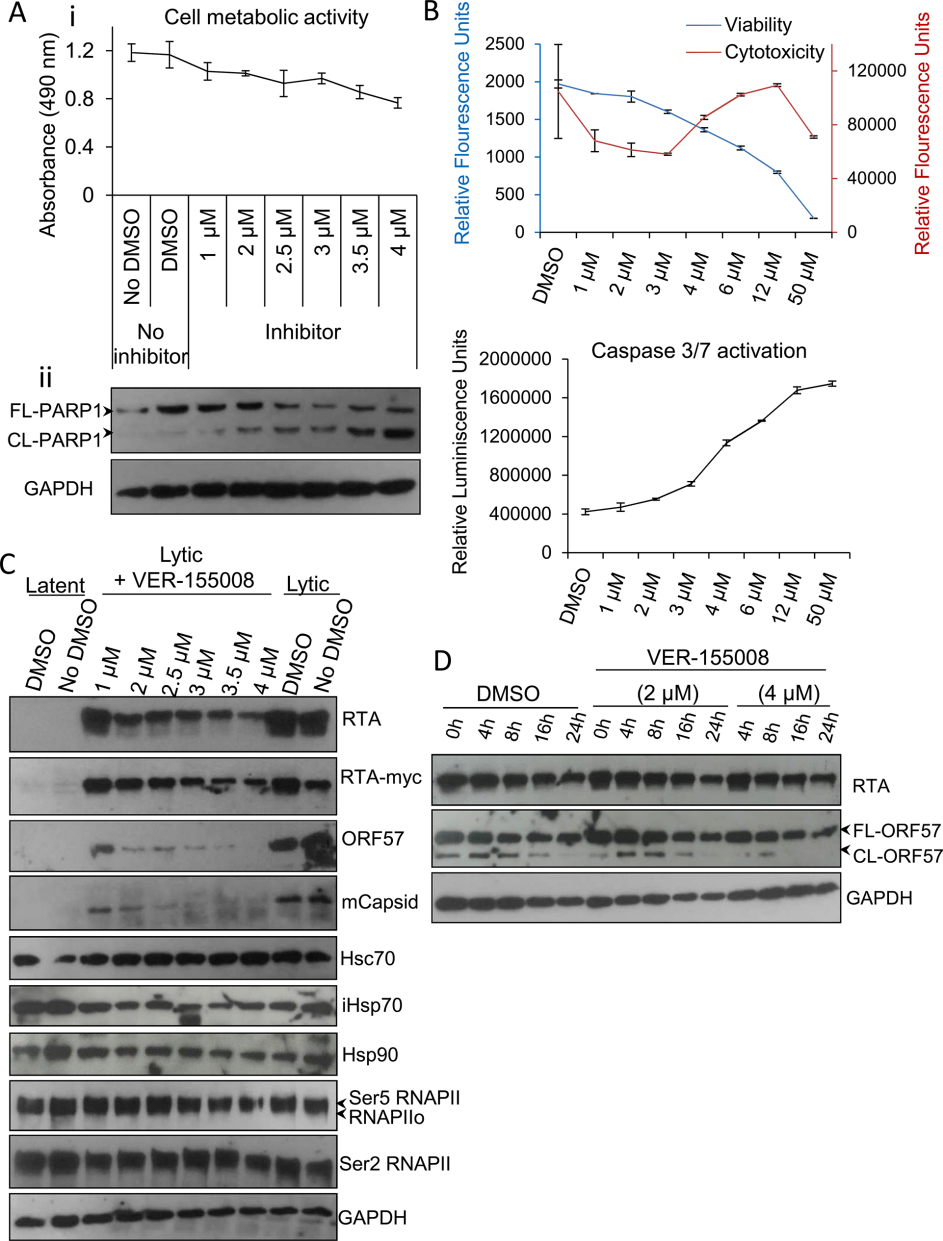
VER-155008 abrogated viral protein synthesis pre-translationally in TREx BCBL1-RTA cells. (A) Cytotoxicity of VER-155008 was assessed in unreactivated cells exposed to increasing inhibitor concentrations for 24 h. (i) Cell metabolic activity was reduced in a dose-dependent manner as quantified using an MTS assay. (ii) A dose-dependent cleavage of full length (FL) PARP1 protein into cleaved (CL) PARP1 was observed in the presence of VER-155008. (B) Unreactivated cells were exposed for 24 h to increasing inhibitor concentrations. Concentrations higher than 3 μM resulted in increased cytotoxicity and activation of effector caspases-3/7 as demonstrated by quantification with ApoTox-Glo Triplex Assay. (C) Immunoblot analysis showing that reactivated cells treated with non-cytotoxic inhibitor concentrations (1 to 2.5 μM) for 24 h revealed a decrease in viral proteins compared with DMSO-treated samples while cellular proteins remained unaffected. RNAPIIo denotes hypophosphorylated RNAPII. Ser5 and Ser2 RNAPII denote serine 5- and serine 2- hyperphosphorylated RNAPII forms respectively. (D) Cells were reactivated for 24 h to allow robust viral protein production. Then, DMSO control (0.1%) or VER-155008 was added in conjunction with cycloheximide (CHX) at 50 μg/ml to block *de novo* protein synthesis. Protein lysates were collected at several times post-CHX addition (0, 4, 8, 16 and 24 h) and analysed by Western blotting. VER-155008 did not alter the half-life of RTA or ORF57 protein, thus these proteins were not clients of Hsp70 isoforms.

Members of the *HSP70* chaperone family possess an N-terminal nucleotide binding domain with ATPase activity which is essential for their function. To examine the implications of Hsc70 and iHsp70 redistribution into KSHV RTCs, a small molecule inhibitor, VER-155008, (a dibenzyl-8-aminoadenosine analog) was utilised. This is the only inhibitor that has been demonstrated to specifically target the highly homologous ATPase pocket present in the three main human Hsp70 isoforms [12, 13, 52, 53], which is highly divergent structurally from the ATPase pocket found in chaperones of the *HSP90* family [12, 54]. As such, VER-155008 functions as an ATP mimetic that specifically inhibits the ATPase activity of members of the *HSP70* family. Initially, cytotoxicity of this compound was assessed in unreactivated TREx BCBL1-RTA cells. Following 24 h inhibitor exposure, using a non-radioactive MTS assay, which quantitatively assesses cell proliferation, a drastic reduction in cell metabolic activity was seen for inhibitor concentrations higher than 6 μM (**S4 Fig**), thus concentrations ranging from 1 to 4 μM were used for further cytotoxicity characterization (**Fig 3Ai**). The inhibitor triggered apoptosis in a dose-dependent manner as demonstrated by the caspase 3-mediated cleavage of full length poly [ADP-ribose] polymerase (FL-PARP1) protein into cleaved PARP1 (CL-PARP1) (**Fig 3Aii**). Small amounts of CL-PARP1 were seen at 1 μM and 2 μM with a significant increase of this form after 3 μM. These results were confirmed with ApoTox-Glo Triplex Assay by quantitatively measuring viability, cytotoxicity and activation of effector caspases-3/7 in the same sample well after 24 h inhibitor treatment. A dose-dependent decrease in viability was evident from 2 μM to 50 μM while cytotoxicity and activation of caspases-3/7 were only considerably increased at concentrations higher than 3 μM (**Fig 3B**).

Next, TREx BCBL1-RTA cells were reactivated for 24 h in the presence of drug vehicle DMSO (0.1%) or a range of increasing inhibitor concentrations. Cells treated with the inhibitor at non-cytotoxic concentrations (1 to 2.5 μM) revealed a drastic reduction in the levels of early ORF57 and late minor capsid (mCapsid) proteins. A moderate reduction in the immediate-early RTA protein was also seen (**Fig 3C**). Of note, when detecting the fusion protein RTA-Myc, which expression is not from the KSHV genome, with anti-Myc antibody, the decrease in RTA-Myc was not as dramatic as that seen for viral RTA, suggesting that the decrease in viral proteins was not due to a general cytotoxic effect of the inhibitor on the cells, and that viral, but not cellular proteins were specifically affected. As an additional cellular control, protein levels of the large subunit of RNAPII, which has a half-life of 12–16 h [55], was assessed with antibodies specific for the different phosphorylated forms of RNAPII. Protein levels of these forms were not significantly changed in the presence of the inhibitor, nor were those of Hsc70 or Hsp90 proteins. Importantly, in the presence of VER-155008, iHsp70 levels were not upregulated. iHsp70 upregulation is a universal hallmark of Hsp90 inhibition not only *in vitro* [56] but also in clinical trials [57], pointing to selectivity for Hsp70 isoforms by VER-155008.

Hsp90 and Hsp70 chaperone machineries have been reported to be crucial for the stability and/or maturation of multiple viral proteins [7, 41, 58–62]. Therefore to ascertain whether Hsp70 isoforms could stabilise the essential KSHV lytic proteins RTA and ORF57, TREx BCBL1-RTA cells were reactivated for 24 h to allow sufficient viral protein expression followed by addition of DMSO control or VER-155008 in conjunction with cycloheximide (CHX) at 50 μg/ml to block *de novo* protein synthesis. Protein lysates were then collected at different times after addition of CHX. The half-life of RTA and ORF57 proteins from inhibitor-treated cells were not altered compared with DMSO-treated cells (**Fig 3D**). These results indicate that the observed decrease in viral protein synthesis was prior to translation and that neither viral RTA nor ORF57 protein were client proteins of the Hsp70 isoforms. As such, this highlights a potentially novel role of Hsp70 isoforms in the KSHV replication cycle independent of viral protein stability and maturation.

**Fig 4:**
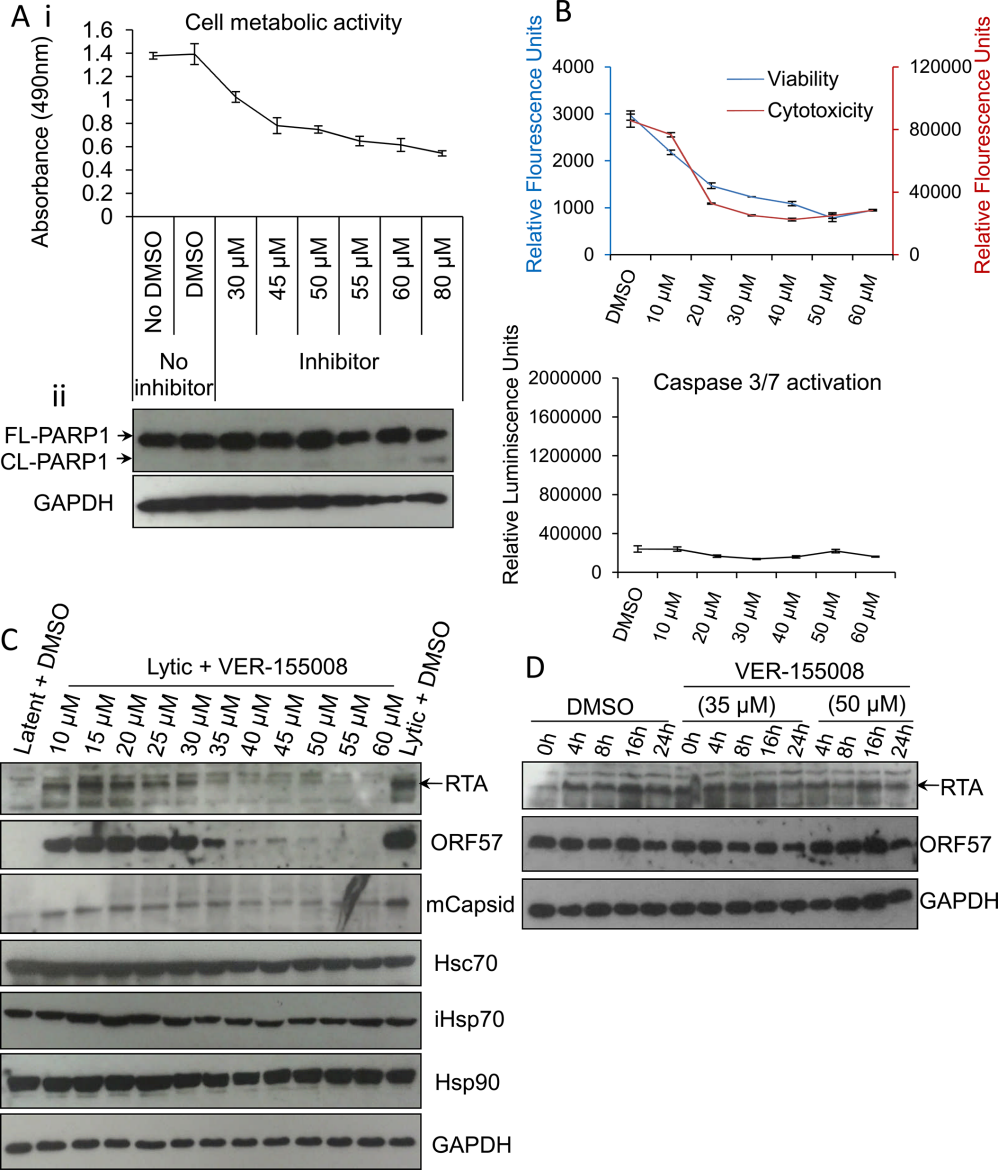
VER-155008 abrogated viral protein synthesis pre-translationally in HEK-293T rKSHV.219 cells. (A) Cytotoxicity of VER-155008 was assessed in unreactivated cells exposed to increasing inhibitor concentrations for 24 h. (i) Cell metabolic activity was reduced in a dose-dependent manner as quantified using an MTS assay. (ii) Treatment with VER-155008 did not readily cause caspase 3-dependent cleavage of PARP1. Only at 80 μM cleaved (CL) PARP1 was observed faintly. (B) Unreactivated cells were exposed for 24 h to increasing inhibitor concentrations. A reduced number of metabolically active cells accompanied by no increased cytotoxicity was observed in the presence of the inhibitor. There was no activation of effector caspases even in the presence of 60 μM VER-155008. This phenotype is consistent with cell cycle arrest. Results were quantified with ApoTox-Glo Triplex Assay. (C) Immunoblot analysis showing that reactivated cells treated with 40 μM VER-155008 for 24 h resulted in abrogation of viral proteins compared with DMSO-treated samples while cellular proteins remained constant. (D) Cells were reactivated for 24 h to allow robust viral protein expression. Then, DMSO control (0.1%) or VER-155008 was added in conjunction with cycloheximide (CHX) at 100 μg/ml to block *de novo* protein synthesis. Protein lysates were collected at several times post-CHX addition (0, 4, 8, 16 and 24 h) and Western blotting was performed. As previously seen in TREx BCBL1-RTA cells, VER-155008 did not alter the half-life of RTA or ORF57 protein.

To further corroborate these results, experiments were also repeated in HEK-293T rKSHV.219 cells. Again, cell metabolic activity, PARP1 cleavage, viability, cytotoxicity and activation of caspases-3/7 in unreactivated cells were all assessed at a range of increasing inhibitor concentrations (**Fig 4A and 4B**). On this occasion, the inhibitor did not trigger apoptosis (**Fig 4Aii and 4B**) but it caused a pronounced cell cycle arrest at 24 h exposure at concentrations of ≥ 20 μM demonstrated by a reduced number of metabolically active cells that exhibited no increased cytotoxicity [63] (**Fig 4B**). It is known that the apoptotic potential of VER-155008 is cell line-dependent and that VER-155008 can cause cell cycle arrest in human colon, breast and lung tumour cell lines [54, 64]. HEK-293T rKSHV.219 cells were also reactivated for 24 h in the presence of drug vehicle DMSO (0.1%) or increasing inhibitor concentrations. Endogenous RTA, ORF57 and mCapsid protein levels were moderately reduced in cells treated at an inhibitor concentration of 10 μM and severely reduced at 40 μM while cellular proteins remain unaffected (**Fig 4C**). These were relatively high inhibitor concentrations compared with TREx BCBL1-RTA cells; nonetheless a concentration of 40 μM has been shown before to be necessary for inhibition of Hsp70 isoforms in human carcinoma cell lines [54]. As previously seen in TREx BCBL1-RTA cells, when blocking *de novo* protein synthesis with CHX at 100 μg/ml in HEK-293T rKSHV.219 cells, the half-life of RTA and ORF57 proteins were not reduced even in the presence of VER-155008 at 50 μM (**Fig 4D**). This supports the findings seen in TREx BCBL1-RTA cells, suggesting that the decrease in viral protein production was due to a pre-translation event.

### The small molecule inhibitor VER-155008 caused a significant reduction in viral transcripts, viral DNA and progeny at non-cytotoxic concentrations

**Fig 5:**
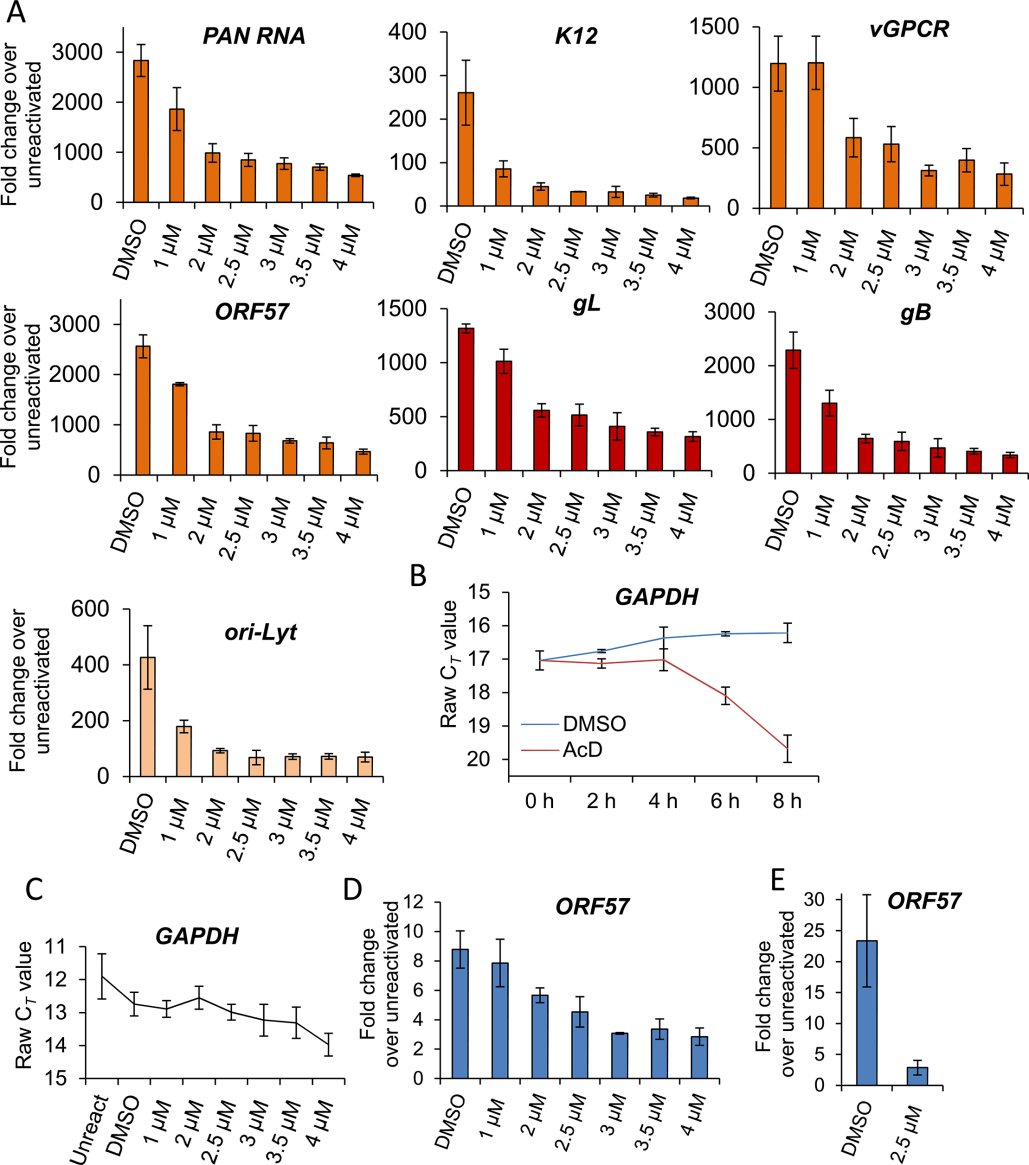
VER-155008 caused a significant reduction in viral transcripts, viral DNA and progeny in TREx BCBL1-RTA cells. (A) Cells were reactivated for 24 h in the presence of control DMSO (0.1%) or a range of increasing inhibitor concentrations, total RNA was isolated and qRT-PCR carried out. Early (orange colour), late (red colour) and *ori-Lyt* viral transcripts were all significantly reduced at 1 μM VER-155008 compared to the levels found in DMSO-treated samples. All samples were normalised to *GAPDH*. Results show the mean of three biological replicates with error bar as standard deviation. (B) The stability of *GAPDH* transcript was assessed in unreactivated TREx BCBL1-RTA cells in the presence of the transcriptional inhibitor actinomycin D (AcD) (2.5 μg/ml) or control DMSO (0.25%). Cells were collected over the time points indicated and total RNA was extracted followed by qRT-PCR. After 6 h AcD treatment, the amount of *GAPDH* transcripts was reduced by more than half (C_*T*_ = 18.09) compared with DMSO-treated cells (C_*T*_ = 16.24). A further reduction was observed at 8 h treatment (C_*T*_ = 19.68). The average of two biological replicates with error bar as standard deviation is shown. (C) The amount of *GAPDH* transcripts did not significantly decrease when using VER-155008 for 24 h at concentrations lower than 3 μM compared to DMSO-treated samples. In contrast, concentrations higher than 3 μM significantly showed a C*_T_* lower than DMSO samples, indicating compromised cellular transcription at these inhibitor concentrations. The same samples in which viral transcripts had been quantified were used to plot all C_*T*_ values. Results show the mean of three biological replicates with error bar as standard deviation. (D) Cells were reactivated for 72 h, total DNA was isolated and real-time qPCR was performed. Viral DNA load was significantly decreased at 2 μM VER-155008. Results show the mean of three biological replicates with error bar as standard deviation. (E) TREx BCBL1-RTA cells were reactivated for 72 h in the presence of VER-155008 at 2.5 μM or vehicle drug DMSO (0.1%). The culture medium was then incubated for 24 h with HEK-293T cells followed by total RNA extraction and qRT-PCR. A significant decrease in viral progeny was seen in inhibitor-treated cells. Results show the mean of three biological replicates with error bar as standard deviation.

**Fig 6:**
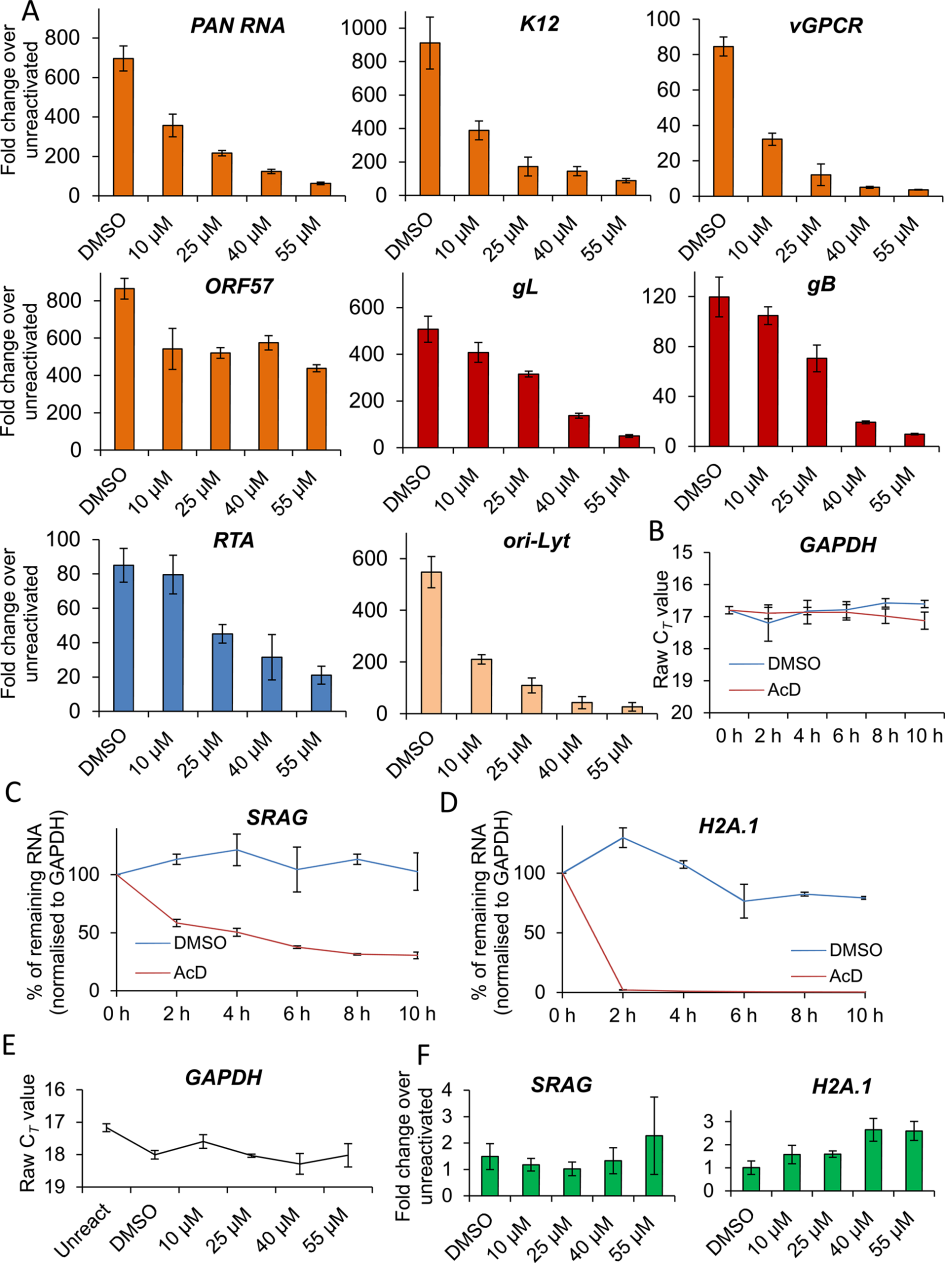
VER-155008 caused a significant reduction in viral transcripts in HEK-293T rKSHV.219 cells. (A) Cells were reactivated for 24 h in the presence of control DMSO (0.1%) or a range of increasing inhibitor concentrations. Quantification by RT-PCR of immediate-early (blue colour), early (orange colour), late (red colour) and *ori-Lyt* viral transcripts showed that all transcripts were significantly decreased at 25 μM VER-155008 compared to the levels found in DMSO-treated samples. All samples were normalised to *GAPDH*. Results show the mean of three biological replicates with error bar as standard deviation. (B-D) The stability of cellular *GAPDH*, *SRAG* and *H2A*.*1* transcripts was determined in mRNA decay assays using actinomycin D (AcD) (10 μg/ml) or control DMSO (1%) in HEK-293T rKSHV.219 cells. Cells were collected over the time points indicated and total RNA was extracted followed by qRT-PCR. The average of two biological replicates with error bar as standard deviation is shown. (B) *GAPDH* transcripts were very stable, with no significant reduction in their levels after 10 h of AcD treatment. (C) At 4 h AcD treatment *SRAG* mRNA levels were reduced by 50% showing a relatively short stability. (D) *H2A*.*1* mRNA levels were very unstable, with nearly 100% reduction after 2 h AcD treatment. (E) *GAPDH* transcript levels remained unchanged following 24 h VER-155008 treatment in reactivated HEK-293T rKSHV.219 cells. The same samples in which viral transcripts had been quantified were used to plot all C_*T*_ values. (F) The unstable cellular *SRAG* and *H2A*.*1* mRNAs were not significantly decreased in the presence of VER-155008 treatment compared with DMSO treatment for 24 h indicating that cellular transcription was not compromised even at high concentrations of VER-155008. The same samples in which viral transcripts had been quantified were used to quantify *SRAG* and *H2A*.*1*. All samples were normalised to *GAPDH*.

As the block in KSHV protein synthesis occurred pre-translationally, viral gene expression was quantified in the absence or presence of VER-155008. TREx BCBL1-RTA cells were reactivated for 24 h and two-step quantitative reverse transcription PCR (qRT-PCR) was carried out to quantify a range of viral transcripts. A significant decrease in early (*PAN*, *ORF57*, *K12* and *vGPCR*), late (*gL* and *gB*) viral transcripts and *ori-Lyt* transcripts was observed in a dose-dependent manner, with all transcripts with the exception of *vGPCR* being significantly reduced at an inhibitor concentration of 1 μM (**Fig 5A**). To determine whether cellular transcription was negatively affected in the presence of VER-155008, firstly the stability of *GAPDH* transcript was determined in mRNA decay assays using the transcriptional inhibitor actinomycin D (AcD) (2.5 μg/ml) in TREx BCBL1-RTA cells. After 6 h of AcD treatment, the amount of *GAPDH* mRNA was reduced by half (**Fig 5B**), indicating a short stability of *GAPDH* mRNA in this cell line. We then plotted the raw cycle threshold (C_*T*_) for *GAPDH* transcript from the same samples in which viral transcripts had been quantified after 24 h of VER-155008 treatment. As the same amount of total RNA was converted into cDNA for all samples, if cellular transcription was not compromised a very similar C*_T_* is expected for all samples. Indeed, samples treated with up to 3 μM VER-155008 were all within 0.4 C*_T_* from the 12.7 C*_T_* of DMSO-treated samples. Only after concentrations higher than 3 μM *GAPDH* mRNA levels were significantly reduced compared to DMSO-treated samples as shown by a significantly higher C_*T*_ value (**Fig 5C**). This is consistent with the cytotoxicity profile of VER-155008 in TREx BCBL1-RTA cells (**Fig 3A and 3B**) and the clear decrease in RTA-myc protein (which expression is not from the KSHV genome) at inhibitor concentrations higher than 3 μM (**Fig 3C**). Taken together, these results suggest that cellular transcription was compromised at concentrations of VER-155008 higher than 3 μM while at concentrations lower than 3 μM transcription was occurring normally. Interestingly, transcription of viral genes which also require host RNAPII for their expression was negatively affected even at VER-155008 concentrations lower than 3 μM. Next, we assessed whether the inhibitor also caused a reduction in viral DNA replication. TREx BCBL1-RTA cells were reactivated for 72 h, total DNA was isolated and real-time qPCR was performed using primers specific for *ORF57*. While DMSO-treated cells reached _~_ 9-fold increase in viral DNA load, inhibitor concentrations of 2 μM or higher resulted in a significant reduction (> 30%) in viral DNA (**Fig 5D**). Moreover, the production of infectious KSHV virions in TREx BCBL1-RTA cells was evaluated in the presence of VER-155008 at 2.5 μM or vehicle drug DMSO. For this, cells were reactivated and treated for 72 h, culture medium was centrifuged and incubated for 24 h with HEK-293T cells. Total RNA was then isolated and qRT-PCR carried out. A significant reduction (_~_ 80%) in the release of infectious viral progeny was observed in inhibitor-treated cells (**Fig 5E**). Viral transcripts were also quantified at 24 h reactivation in HEK-293T rKSHV.219 cells in the absence or presence of VER-155008. At non-cytotoxic concentrations of 10 μM there was a drastic decrease for all early viral transcripts and *ori-Lyt* transcripts (**Fig 6A**). It is intriguing that *ORF57* mRNA levels did not show a clear dose-response with the inhibitor as seen for *ORF57* mRNA in TREx BCBL1-RTA cells; however the levels were decreased compared with DMSO-control cells. This is the only transcript of all the viral transcripts tested in both cell lines that did not show a dose-response. However, Western blotting did reveal a complete reduction of ORF57 protein in cells treated with > 40 μM VER-155008 (**Fig 4C**). This suggests that Hsp70 isoforms may also play a role in the folding of ORF57 protein. In fact, Hsc70 has been previously reported to associate with at least 15–20% of newly synthesized proteins during their biogenesis [65], thus, a role for Hsp70 isoforms in folding viral proteins cannot be ruled out. In order to evaluate cellular transcription activity in the presence of VER-155008, we first determined *GAPDH* transcript stability in mRNA decay assays using AcD (10 μg/ml) in HEK-293T rKSHV.219 cells. In contrast to TREx BCBL1-RTA cells, *GAPDH* transcripts were very stable, with no significant reduction in their levels after 10 h of AcD treatment (**Fig 6B**). The half-lives of two cellular transcripts, *SRAG* (a cellular mRNA export adapter) and histone H2A (*H2A*.*1*) were also determined in HEK-293T rKSHV.219 cells. *SRAG* transcripts were reduced to 50% following 4 h AcD treatment (**Fig 6C**), while the *H2A*.*1* mRNA was very unstable with nearly 100% reduction after 2 h AcD treatment (**Fig 6D**). We then plotted the raw C_*T*_ values for *GAPDH* transcript from samples in which viral transcripts had been quantified after 24 h of VER-155008 treatment; these did not significantly change in the presence of VER-155008 (**Fig 6E**). Next, the unstable *SRAG* and *H2A*.*1* mRNAs were measured in the same samples in which viral transcripts had been quantified after 24 h of VER-155008 treatment. In contrast to viral transcripts, both cellular transcripts were not significantly reduced, indicating that transcription of cellular genes was occurring normally even in the presence of high VER-155008 concentrations (**Fig 6F**). Taken together, these results demonstrate that inhibition of Hsp70 isoform function abrogated the expression of viral genes from various temporal classes; however cellular RNAPII-mediated transcription was not compromised when using VER-155008 at non-cytotoxic concentrations.

### Hsp70 isoforms are not required for RTA-mediated transactivation of KSHV promoters

**Fig 7:**
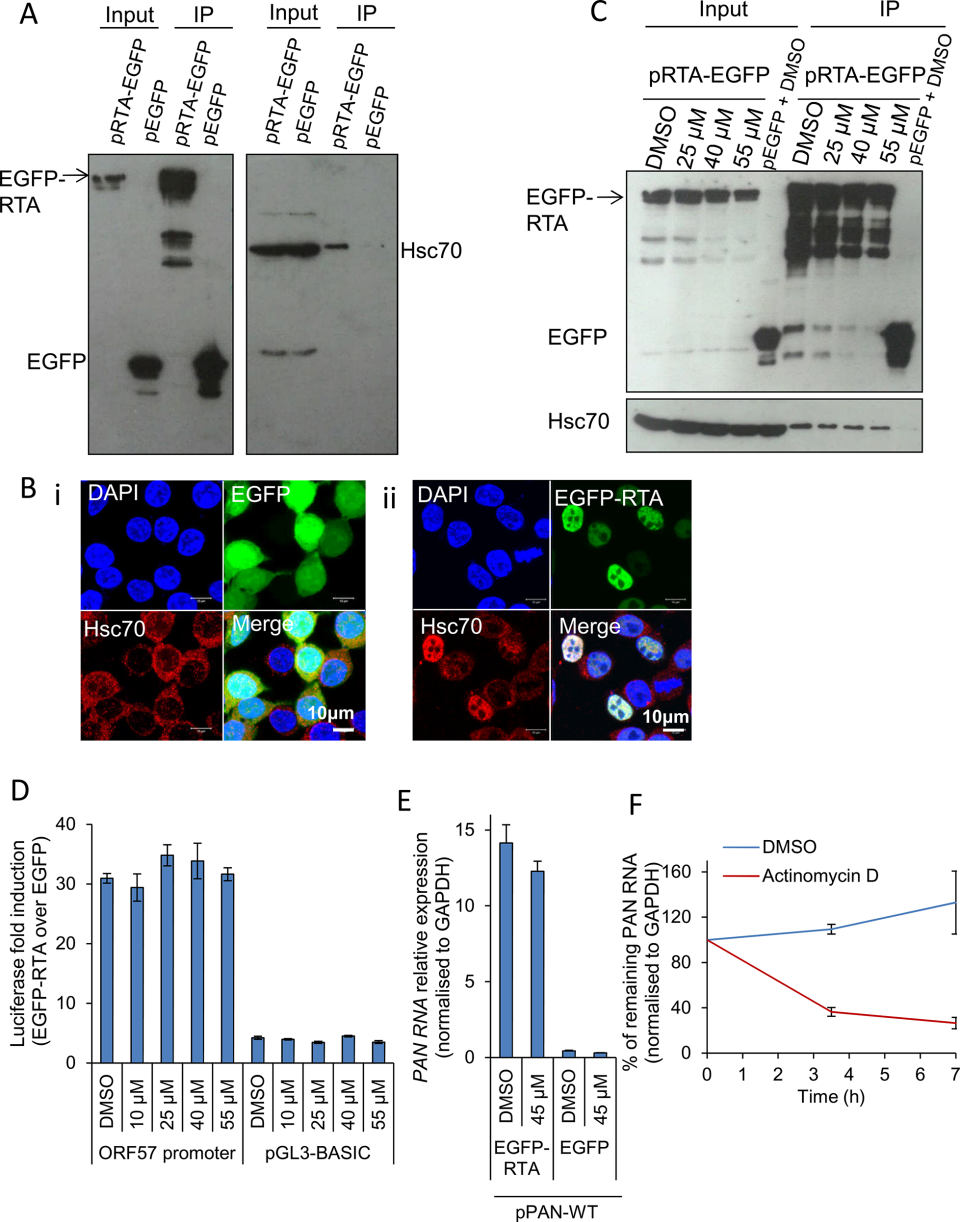
Hsp70 isoforms were not required for RTA-mediated transactivation of KSHV promoters. (A) HEK-293T cells were transiently transfected with control pEGFP or pRTA-EGFP. 24 h post-transfection immunoprecipitations (IP) were carried out using a GFP-specific antibody. Endogenous Hsc70 interacted with EGFP-RTA but not with the control protein EGFP. (B) HEK-293T cells were transfected with control pEGFP or pRTA-EGFP for 24 h and then analysed by immunofluorescence. Endogenous Hsc70 was cytoplasmic in cells expressing EGFP (i). In contrast, Hsc70 strongly co-localised with EGFP-RTA in the nucleus, but not the nucleoli (ii). (C) HEK-293T cells were transiently transfected with control pEGFP or pRTA-EGFP. 24 h post-transfection, VER-155008 was added and incubated for a further 24 h. Then, immunoprecipitations were carried out using a GFP-specific antibody. Even in the presence of 55 μM VER-155008 the interaction between Hsc70 and EGFP-RTA was not disrupted. (D) HEK-293T cells were co-transfected with pRTA-EGFP along with the *Renilla* luciferase vector and either the *ORF57* promoter firefly luciferase reporter vector, or the empty reporter vector (pGL3-BASIC). The same co-transfections were performed using pEGFP as a negative control protein. 24 h post-transfection, cells were exposed for 2 h to VER-155008 and luciferase activities were measured. Comparable activation of the RTA-responsive *ORF57* promoter to that seen in DMSO-treated cells occurred in cells treated with 55 μM VER-155008. The results of three independent transfections were averaged with error bars as standard deviation. (E) HEK-293T cells were transiently co-transfected with pPAN-WT and either pRTA-EGFP or control pEGFP. 24 h post-transfection either vehicle drug DMSO (0.1%) or 45 μM VER-155008 was added and incubated for a further 24 h. Total RNA was then extracted and qRT-PCR performed. RTA-mediated promoter transactivation and subsequent synthesis of *PAN* RNA occurred at a similar rate in the presence of VER-155008 or control DMSO. The results of three independent transfections were averaged with error bars as standard deviation. (F) The stability of *PAN* RNA was determined in HEK-293T cells that had been co-transfected with pPAN-WT and pRTA-EGFP. Following 24 h post-transfection, actinomycin D (AcD) (5 μg/ml) or DMSO control (0.5%) was added. Cells were collected over the time points indicated and total RNA was extracted followed by qRT-PCR. The average of two biological replicates with error bar as standard deviation is shown.

Following quantification of viral transcripts in both cell lines, it appeared that the reduction seen in viral gene expression, protein production and infectious virion production could be a consequence of a significant global reduction of viral transcripts in inhibitor-treated cells. This led to the possibility that Hsp70 isoforms could be implicated in activation of viral promoters and subsequent transcription or alternatively Hsp70 isoforms were required for KSHV RTC formation. Because viral RTA and Hsc70 co-localized in TREx BCBL1-RTA cells and RTA is the master latent-lytic transactivator for multiple KSHV immediate-early, delayed-early and late promoters [66–70], we further investigated the possibility that Hsc70 was required for RTA-mediated transactivation. Initially we assessed whether an interaction occurred between Hsc70 and RTA in the absence of other viral proteins or DNA. For this, HEK-293T cells were transiently transfected for 24 h with control pEGFP or pRTA-EGFP and immunoprecipitations were carried out using a GFP-specific antibody. RTA-EGFP precipitated endogenous Hsc70 in contrast to the control EGFP protein (**Fig 7A**). In addition, HEK-293T cells were transfected with control pEGFP or pRTA-EGFP for 24 h and examined by immunofluorescence. In cells expressing EGFP protein, endogenous Hsc70 remained cytoplasmic (**Fig 7Bi**), while in EGFP-RTA-expressing cells Hsc70 strongly co-localised with RTA in the nuclei, suggesting RTA expression alone is sufficient to redistribute Hsc70 into the nucleus (**Fig 7Bii**). Similar nuclear redistribution was also seen for endogenous iHsp70 (**S5 Fig**). Next, we determined whether VER-155008 was able to disrupt the interaction between EGFP-RTA and Hsc70 in HEK-293T cells. HEK-293T cells exhibited a very similar cytotoxicity profile to that seen in HEK-293T rKSHV.219 cells (**S6 Fig**). HEK-293T cells were transiently transfected with pRTA-EGFP or control pEGFP. To allow maximal protein expression and avoid interference of the inhibitor with the transfection, the inhibitor was added at 24 h post-transfection and incubated for a further 24 h, prior to immunoprecipitations being performed. Western blot analysis revealed that the inhibitor did not disrupt the interaction between Hsc70 and RTA even at high inhibitor concentrations (55 μM) (**Fig 7C**), suggesting that the ATPase function of Hsc70 is not required for the interaction with RTA protein.

Therefore, to investigate whether Hsc70 was required for RTA-mediated transactivation of the RTA-responsive *ORF57* promoter, a dual-luciferase reporter assay system was utilised. HEK-293T cells were co-transfected with pRTA-EGFP along with the *Renilla* luciferase vector and either the *ORF57* promoter firefly luciferase reporter vector, or the empty reporter vector (pGL3-BASIC). The same co-transfections were performed using pEGFP, as a negative control. 24 h post-transfection, cells were exposed for 2 h to increasing concentrations of VER-155008 and luciferase activities were measured. Longer exposure times to the inhibitor affected the formation of the control *Renilla* luciferase protein which has a half-life of 3 h, suggesting that Hsp70 isoforms may be required for the folding/maturation of this enzyme. In the presence of EGFP-RTA and the *ORF57* promoter reporter construct, the *ORF57* promoter activity was increased _~_ 30-fold, while the empty vector had a _~_ 4-fold increase. However, *ORF57* promoter activity was not significantly decreased in the presence of VER-155008 (**Fig 7D**). To confirm this result, HEK-293T cells were also transiently co-transfected with pPAN-WT, a plasmid encoding the genomic region of wild type *PAN* RNA including its promoter region [71], and either pRTA-EGFP or control pEGFP. 24 h post-transfection either vehicle drug DMSO or VER-155008 at 45 μM was added and incubated for a further 24 h followed by qRT-PCR. In the presence of EGFP-RTA, but not of control EGFP protein, *PAN* RNA was synthesised. However, no significant decrease in the amount of *PAN* RNA was seen in inhibitor-treated cells (**Fig 7E**), indicating that RTA-mediated promoter transactivation and subsequent synthesis of *PAN* RNA was occurring normally in the presence of VER-155008. These data demonstrate that Hsp70 isoforms did not directly enhance RTA-mediated transactivation. To assess the transcription activity of cellular RNAPII in the presence of the inhibitor, the half-life of *PAN* RNA was determined in HEK-293T cells co-transfected with pPAN-WT and pRTA-EGFP. Following 24 h post-transfection, AcD (5 μg/ml) or DMSO control (0.5%) was added. After 7 h of transcription inhibition, *PAN* RNA levels were reduced to 25% compared to DMSO-treated cells (**Fig 7F**). This quick reduction in the stability of *PAN* RNA in the absence of ORF57 protein is in agreement with previous reports [72]. Thus, if VER-155008 was blocking general RNAPII transcription, a significant reduction in *PAN* RNA levels should be observed after 7 h of inhibitor treatment; however, *PAN* RNA levels were not reduced in cells treated with VER-155008 for 24 h (**Fig 7E**).

### Inhibition of Hsp70 isoforms abrogated KSHV RTCs formation and RNAPII re-localization to viral promoters

**Fig 8:**
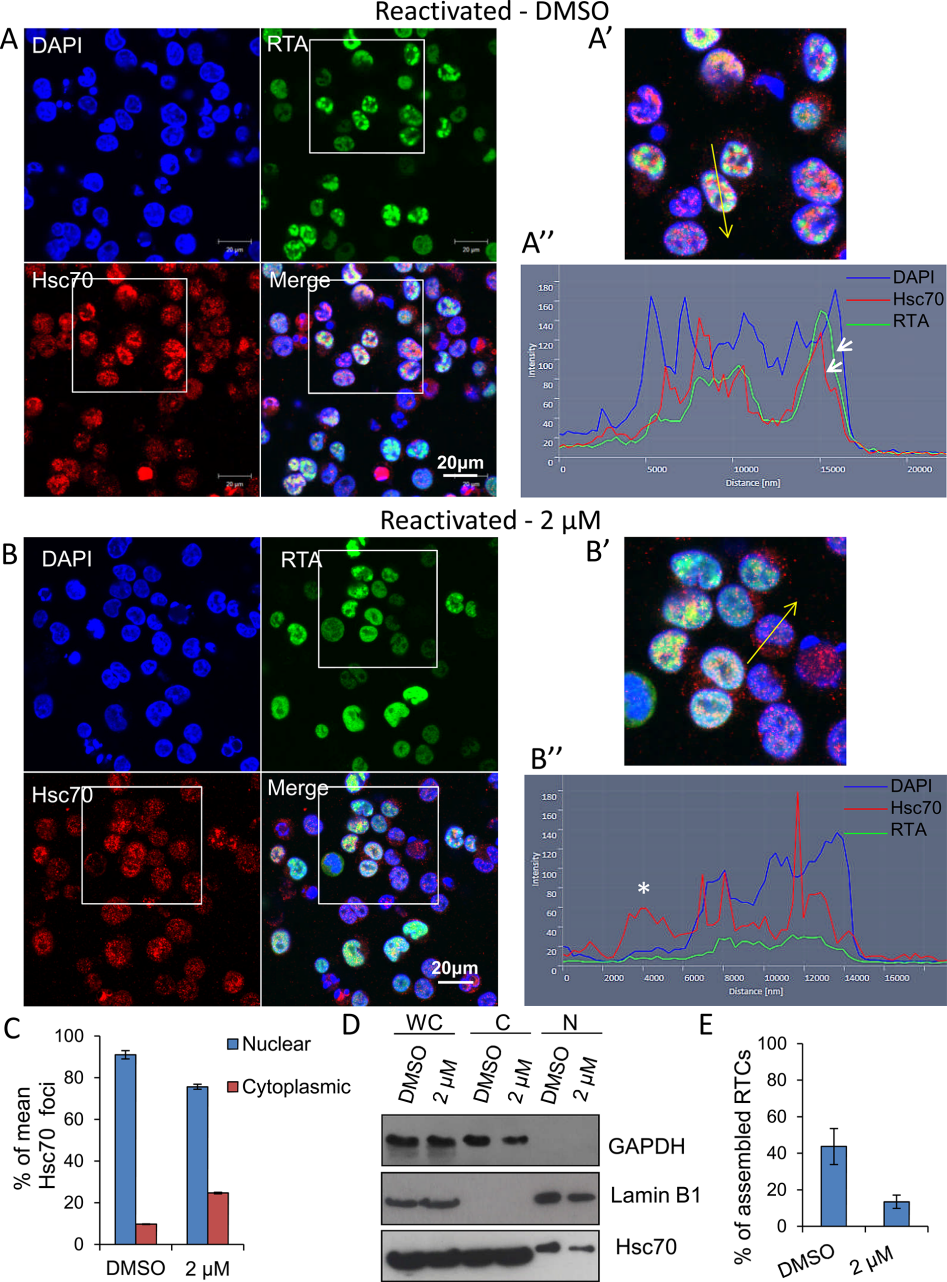
Inhibition of Hsp70 isoforms prevented Hsc70 chaperone recruitment to KSHV RTCs and RTC formation. (A) TREx BCBL1-RTA cells were reactivated and treated with control DMSO (0.1%) for 24 h. Multiple KSHV RTCs identified by RTA-labelling were formed in DMSO-treated cells to which large and numerous Hsc70 foci were recruited. In these cells, Hsc70 was depleted from the cytoplasm. (A’) Confocal images were subjected to profiling analysis using Zeiss Zen 2011 software. Profiling was conducted for each cell in two representative confocal images taken with a 40-times objective; this included profiling a total of 49 DMSO-treated cells. A representative line profile (yellow line) and accompanying relative intensities of each channel at every pixel along the line is shown for a DMSO-treated reactivated cell. The DAPI intensity profile shows values below 25 outside the nucleus boundary. RTA profile shows a peak with intensity of 160 that lies within the DAPI boundaries corresponding to a KSHV replication compartment. Hsc70 peaks lie within the DAPI boundaries demonstrating their nuclear location. An Hsc70 intensity peak closely resembles the RTA intensity peak (arrows), indicating co-localization of Hsc70 with RTA since both peaks occur at the same position along the line. (B) In cells reactivated and treated with 2 μM VER-155008 for 24 h, RTA was diffuse in the nucleus and not assembled into KSHV RTCs. In addition, Hsc70 nuclear foci were smaller and less abundant than in DMSO-treated cells and Hsc70 was not depleted from the cytoplasm. (B’). Confocal profiling was performed for each cell in two representative confocal images taken with a 40-times objective; a total of 68 inhibitor-treated cells were analysed. A representative line profile (yellow line) is shown for an inhibitor-treated cell. An Hsc70 peak is seen outside the DAPI boundaries corresponding to an Hsc70 cytoplasmic peak (asterisk). (C) Total Hsc70 peaks and total nuclear Hsc70 peaks per cell were combined for each confocal image and experimental condition. Data was converted to percentage of nuclear and cytoplasmic peaks. From this mean percentage, standard error of the mean was calculated. (D) Reactivated TREx BCBL1-RTA cells for 24 h in the presence or absence of 2 μM VER-155008 were fractionated into whole cell (WC), cytoplasmic (C) and nuclear (N) fractions. Equal amounts of total protein from each fraction were analysed by Western blotting. A slight increase in cytoplasmic Hsc70 which correlated with a small decrease in nuclear Hsc70 was detected in inhibitor-treated cells. (E) The percentage of assembled RTCs in the presence and absence of 2 μM VER-155008 for 24 h was calculated by counting all the cells and corresponding assembled RTCs present in six representative confocal images taken with a 40-times objective. A total of 227 cells were counted in DMSO-treated cells and a total of 260 cells in inhibitor-treated cells. DMSO-treated cells had _~_ 44% assembled RTCs, in contrast, in the presence of VER-155008, only _~_ 13% of the cells presented well-developed RTCs.

A dramatic reduction in early, late and *ori-Lyt* transcripts after 24 h treatment with VER-155008 was evident during KSHV infection in both cell lines used. However, Hsp70 isoforms were not required for RTA-mediated transactivation of KSHV promoters in transiently transfected cells. Thus, we next monitored KSHV RTC formation in the absence or presence of the inhibitor during KSHV lytic infection. TREx BCBL1-RTA cells were reactivated and treated with either control DMSO or 2 μM inhibitor. At 24 h reactivation cells were fixed and immunofluorescence was performed using RTA- and Hsc70-specific antibodies. DMSO-treated cells displayed abundant RTCs and numerous nuclear Hsc70 foci that partially co-localised with RTCs. Hsc70 cytoplasmic depletion was also observed (**Fig 8A and 8A’**). In contrast, inhibitor-treated cells showed diffuse nuclear RTA that was not able to assemble into RTCs (**Fig 8B and 8B’**). In these cells, Hsc70 nuclear foci were still visible, but these were much less numerous and smaller compared with the foci seen in DMSO-treated cells. Significantly, following VER-155008 treatment, Hsc70 was observed in the cytoplasm of reactivated cells (**Fig 8B and 8B’**). Hsc70 subcellular localization was also analysed in DMSO- and inhibitor-treated cells by confocal profiling. Profiling was performed by drawing a line long enough (_~_ 20 μm) to cover the nucleus and cytoplasm at either side of the nucleus. If an Hsc70 pixel intensity data point was equal or greater than to the data point in the previous and subsequent pixel and above the background noise, it was considered as an Hsc70 peak, representing an Hsc70 foci. DMSO-treated cells predominantly showed Hsc70 peaks only within the DAPI boundaries, that is, within the nucleus (**Fig 8A”**). Inhibitor-treated cells displayed Hsc70 peaks outside the DAPI boundaries, that is, in the cytoplasm (**Fig 8B” asterisk**) more often than control cells. A significant increase in cytoplasmic Hsc70 peaks was seen in inhibitor-treated cells compared with DMSO control cells (**Fig 8C**). Fractionation of reactivated TREx BCBL1-RTA cells in the presence or absence of VER-155008, also pointed to slightly higher levels of Hsc70 in the cytoplasm and a decrease in nuclear Hsc70 in inhibitor-treated cells compared with DMSO control cells (**Fig 8D**). Cells were also labelled with Click-iT EdU Alexa Fluor 647 and an antibody specific for Hsc70 (**S7 Fig**). The percentage of assembled RTCs in DMSO- and inhibitor-treated cells was also calculated. In DMSO-treated cells _~_ 44% of cells presented assembled RTCs while only 13% of inhibitor-treated cells showed assembled RTCs (**Fig 8E**). This demonstrates that chaperone recruitment to the nucleus is essential for the assembly of KSHV RTCs and treatment with VER-155008 was sufficient to impair nuclear chaperone recruitment and KSHV RTC formation.

**Fig 9:**
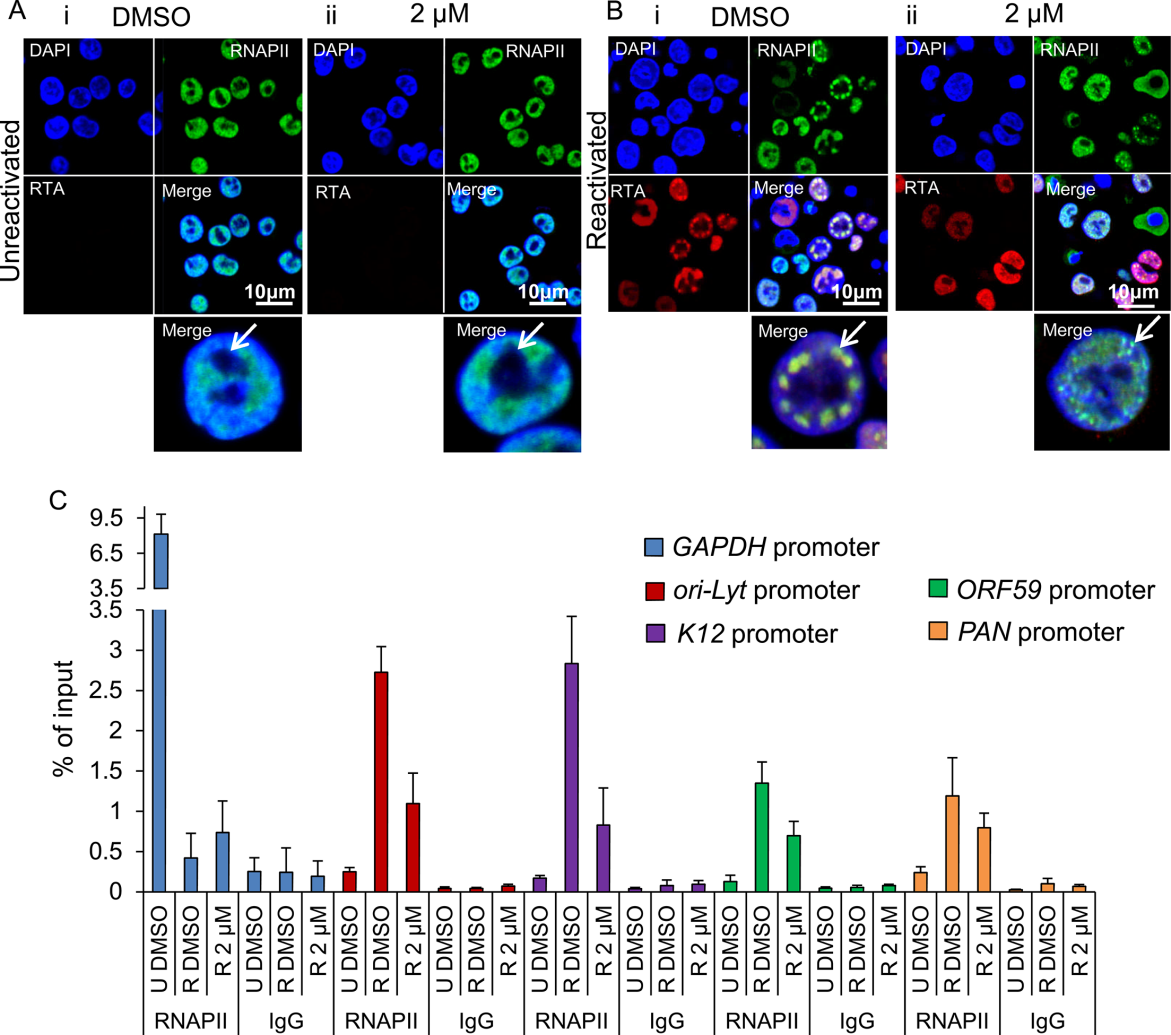
Inhibition of Hsp70 isoforms curtailed RNAPII relocation to KSHV RTCs. (A) TREx BCBL1-RTA cells remained unreactivated and treated with control DMSO (0.1%) (i) or 2 μM VER-155008 (ii). A polyclonal antibody against RTA and a monoclonal antibody (CTD4H8) against RNAPII were used for immunoflourescence analysis. RNAPII protein was nuclear excluding the nucleoli (arrows) regardless of inhibitor treatment. (B) TREx BCBL1-RTA cells were reactivated and treated with either control DMSO (0.1%) or 2 μM VER-155008 for 24 h. In DMSO-treated cells RNAPII and RTA were recruited to viral RTCs (i arrow) while in inhibitor-treated cells RTA was diffuse in the nucleus and RNAPII formed numerous small foci that excluded the nucleoli and resembled pre-replicative sites (ii arrow). (C) TREx BCBL1-RTA cells were either reactivated (R) in the presence of DMSO (0.1%) or 2 μM VER-155008 for 24 h. Unreactivated (U) cells treated with DMSO (0.1%) were used to assess levels of lytic reactivation. ChIP assays were carried out with either monoclonal CTD4H8 RNAPII antibody or mouse control antibody (IgG). In the presence of VER-155008, significantly reduced amounts of RNAPII bound to viral promoters were detected. The average of three independent experiments is shown with error bars as standard deviation.

The subcellular localisation of RNAPII was also assessed by indirect immunoflourescent labelling in TREx BCBL1-RTA cells with the monoclonal antibody CTD4H8, which specifically recognises unphosphorylated and serine-5 phosphorylated RNAPII. In unreactivated cells, RNAPII exhibited a nuclear localization, excluding the nucleolus (**Fig 9Ai arrow**) irrespective of the presence of DMSO (**Fig 9Ai**) or the inhibitor (**Fig 9Aii**). However, in reactivated and DMSO-treated cells, RNAPII was clearly hijacked to RTCs (**Fig 9Bi**). In contrast, in reactivated cells treated with the inhibitor, RNAPII was diffuse throughout the nucleus, but excluding the nucleoli, and formed very small foci (**Fig 9Bii arrow**) (see higher magnification on **S8 Fig**). This suggests that in the presence of Hsp70 isoform inhibition, RNAPII failed to assemble into developed RTCs and instead aberrantly formed what resembled pre-replicative sites. Cells were also labelled with Click-iT EdU Alexa Fluor 647 and an antibody specific for RNAPII (**S9 Fig**). Similar RNAPII subcellular localisation and cellular DNA replication levels were seen in DMSO-treated and 2 μM inhibitor-treated unreactivated cells (**S9Ai and S9Aii Fig respectively**). In DMSO-reactivated cells, cell cycle arrest was evident as shown by fewer Edu-labelled cells (**S9Bi Fig**) consistent with previous reports that lytic KSHV in primary effusion lymphoma cell lines causes G_1_ cell cycle arrest [73]. DMSO-treated cells displayed well assembled RTCs, with few of them showing actively replicating viral DNA (**S9Bi Fig**). Inhibitor-treated cells exhibited very small RNAPII foci and diffuse nuclear Edu labelling (**S9Bii Fig**). To confirm that inhibition of Hsp70 isoform function was able to abolish RNAPII recruitment to viral genomes, we also utilised chromatin immunoprecipitation (ChIP) assays in TREx BCBL1-RTA cells that were either reactivated for 24 h in the presence of DMSO or 2 μM VER-155008 (**Fig 9C**). In unreactivated control cells, there was a clear enrichment of RNAPII at the promoter of *GAPDH* gene, while RNAPII occupancy at viral promoters was minimal. However, KSHV reactivation in DMSO-treated cells led to a drastic reduction of RNAPII at the promoter of *GAPDH* and a significant increase of RNAPII at the viral promoters in agreement with the previous immunofluorescence results, showing RNAPII recruitment to RTCs (**Fig 9Bi**). Conversely, upon treatment of the *HSP70* inhibitor, the amount of RNAPII bound at the promoters of *ori-Lyt*, *K12 and ORF59* was decreased by _~_ 65%, _~_ 70% and _~_ 50% respectively compared with DMSO-treated reactivated cells. Importantly, these results indicate for the first time that inhibition of Hsp70 isoforms leads to a severe impairment in RNAPII recruitment at multiple viral promoters including that of *ori-Lyt*.

### Depletion of Hsc70 reduced KSHV lytic transcription

**Fig 10:**
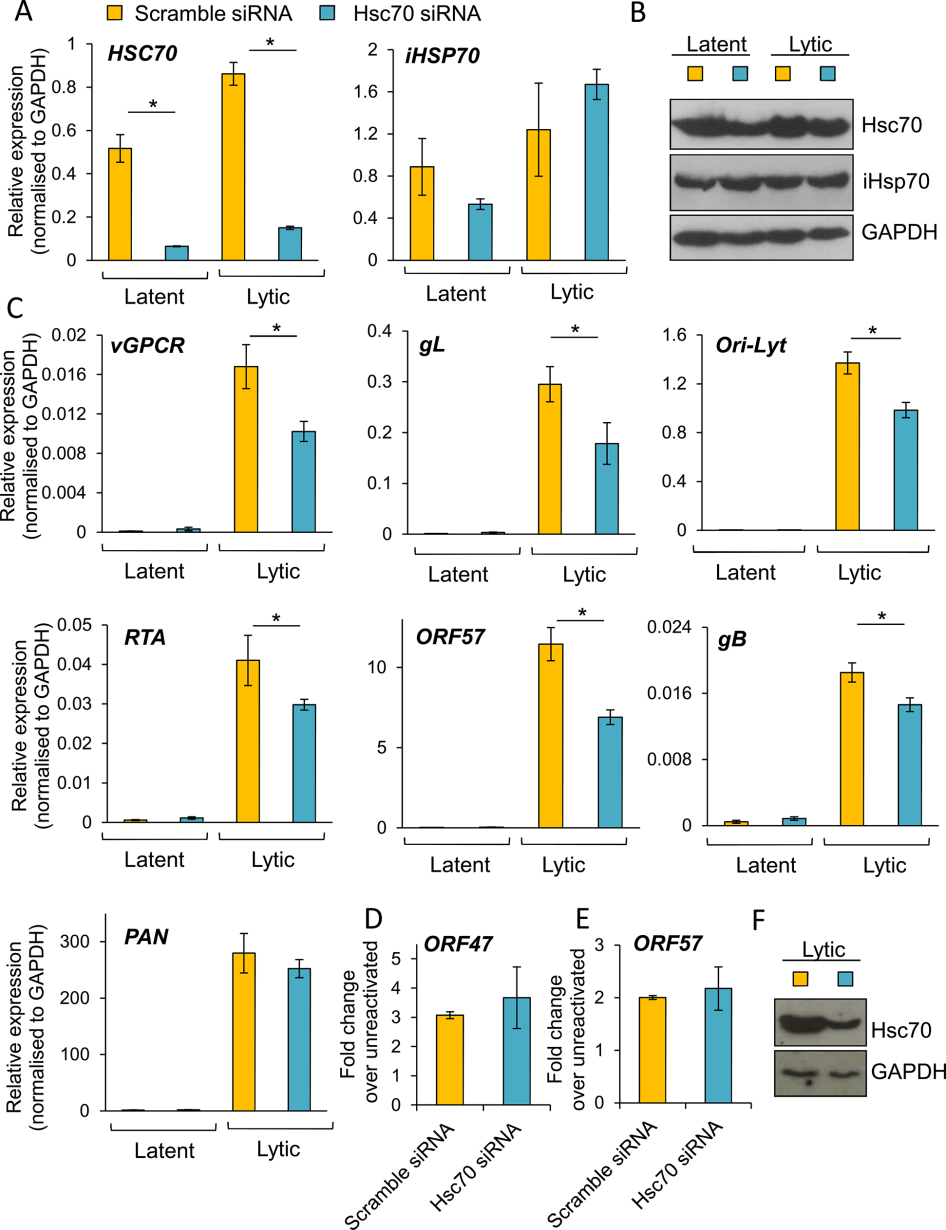
Specific depletion of Hsc70 significantly reduced KSHV lytic transcription in HEK-293T rKSHV.219 cells. Cells were transfected twice with 100 nM Hsc70-specific siRNA or 100 nM scramble siRNA. Following four days post-siRNA transfection, cells were either reactivated for 24 h or remained unreactivated and total RNA and protein were extracted from the same sample. Despite achieving _~_ 85% Hsc70 knockdown at the mRNA level (A), significant amounts of Hsc70 protein remained in Hsc70 siRNA-treated samples (B). Despite this small knockdown at the protein level, in Hsc70-depleted cells there was a significant decrease in the amount of multiple viral transcripts from various temporal classes as quantified by RT-PCR analysis (C). Viral DNA replication was assessed by qPCR following Hsc70-knockdown. Cells were reactivated for 72 h after four days post-siRNA transfection. No significant differences were observed between scramble and Hsc70-treated cells (D). Similar virion production was detected in scramble and Hsc70-treated cells. Cells were reactivated for 72 h after four days post-siRNA transfection, culture medium was centrifuged and immediately incubated with HEK-293T cells for 24 h. Total RNA was then isolated and qRT-PCR carried out (E). Incomplete Hsc70-knockdown at the protein level was observed by Western blotting even after seven days post-siRNA transfection in HEK-293T rKSHV.219 cells. This demonstrates the remarkable stability of Hsc70 in this cell line (F). The average of three independent transfections is shown with error bars as standard deviation (A, C-E).

**Fig 11:**
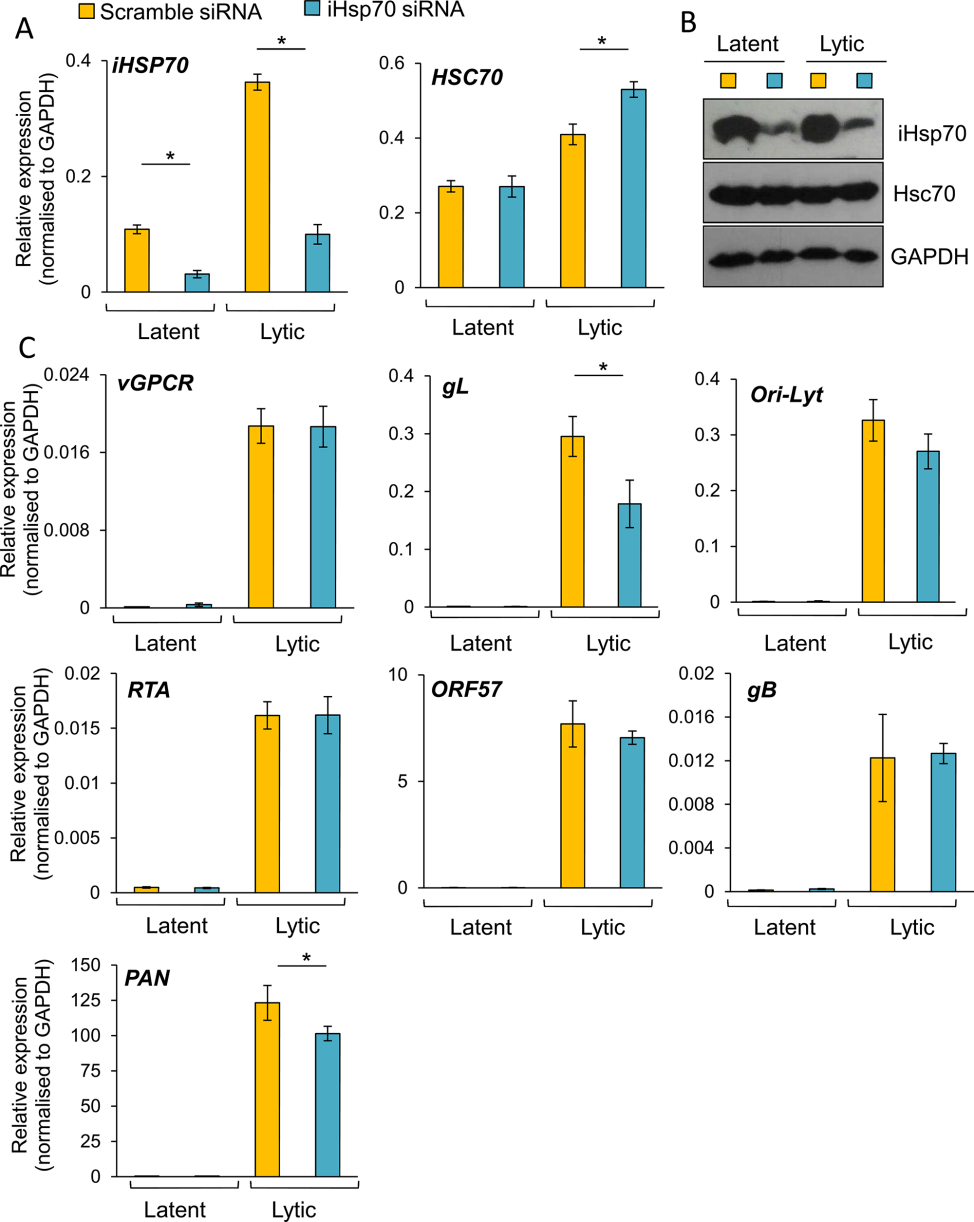
The majority of KSHV lytic gene expression was unaffected following specific depletion of iHsp70 in HEK-293T rKSHV.219 cells. Cells were transfected twice with 100 nM iHsp70-specific siRNA or 100 nM scramble siRNA. Following four days post-siRNA transfection, cells were either reactivated for 24 h or remained unreactivated and total RNA and protein were extracted from the same sample. Efficient iHsp70 knockdown was achieved both at the mRNA level (_~_ 75%) (A) and the protein level (B). A small decrease in the amount of *gL* and *PAN* transcripts was observed in iHsp70-depleted cells (C). The average of three independent transfections is shown with error bars as standard deviation.

To further confirm the essential role of Hsc70 and iHsp70 in the formation of KSHV RTCs, specific individual siRNA-mediated depletion of both isoforms was performed in HEK-293T rKSHV.219 cells. Following four days post-siRNA transfection, cells were reactivated for 24 h and RNA and protein were extracted from the same sample. Hsc70 depletion was evaluated by Western blotting and by qRT-PCR, the latter showing _~_ 85% *Hsc70* mRNA knockdown (**Fig 10A**). In contrast, *iHsp70* mRNA levels were not affected confirming specificity of the Hsc70 siRNA. Despite a successful knockdown at the mRNA level, significant amounts of Hsc70 protein remained in Hsc70-depleted cells (**Fig 10B**). However, even with this modest amount of depletion at the protein level, all viral transcripts (with the exception of *PAN*) displayed a significant reduction in Hsc70 siRNA-treated samples compared with the scramble siRNA-treated cells as demonstrated by qRT-PCR analysis (**Fig 10C**). *ORF57*, *ORF74* and *gL* mRNA levels were decreased by _~_ 40% following Hsc70 knockdown. *Ori-Lyt* and *RTA* transcripts were reduced by _~_ 30% and *gB* levels by _~_ 20%. This suggests that depletion of Hsc70 impaired the expression of viral genes from various temporal classes and thus Hsc70 may be necessary for KSHV RTC formation. Viral DNA replication was also assessed following Hsc70 knockdown. For this, after four days post-siRNA transfection, cells were reactivated for a further 72 h. There were no significant differences between scramble and depleted cells (**Fig 10D**). The production of infectious KSHV virions was also evaluated after 72 h reactivation. Again, no significant differences were seen between scramble and depleted cells (**Fig 10E**); however this result is not surprising due to incomplete Hsc70 depletion even after seven days post-transfection (**Fig 10F**). This highlights the remarkable stability of Hsc70 protein in this cell line and it suggests that Hsc70 depletion was enough to cause a reduction in viral transcripts but not enough to cause a reduction in the amount of viral proteins, thus KSHV lytic replication remained unaffected. It is also possible that iHsp70 was able to functionally compensate for Hsc70. Next, specific depletion of iHsp70 was performed in HEK-293T rKSHV.219 cells. iHsp70 depletion at the mRNA level reached _~_ 75% knockdown (**Fig 11A**) which correlated with efficient depletion at the protein level (**Fig 11B**). However, in iHsp70-depleted cells, the majority of viral gene expression was unaffected, apart from *gL* and *PAN* transcripts which were decreased by _~_ 40% and _~_ 20% respectively (**Fig 11C**). Importantly, taken together these results indicate that partial depletion of Hsc70 at the protein level is sufficient to cause a reduction in viral transcription, suggesting an essential role of this chaperone in the formation of KSHV RTCs, whereas iHsp70 may have a more subtle effect on viral gene expression.

**Fig 12:**
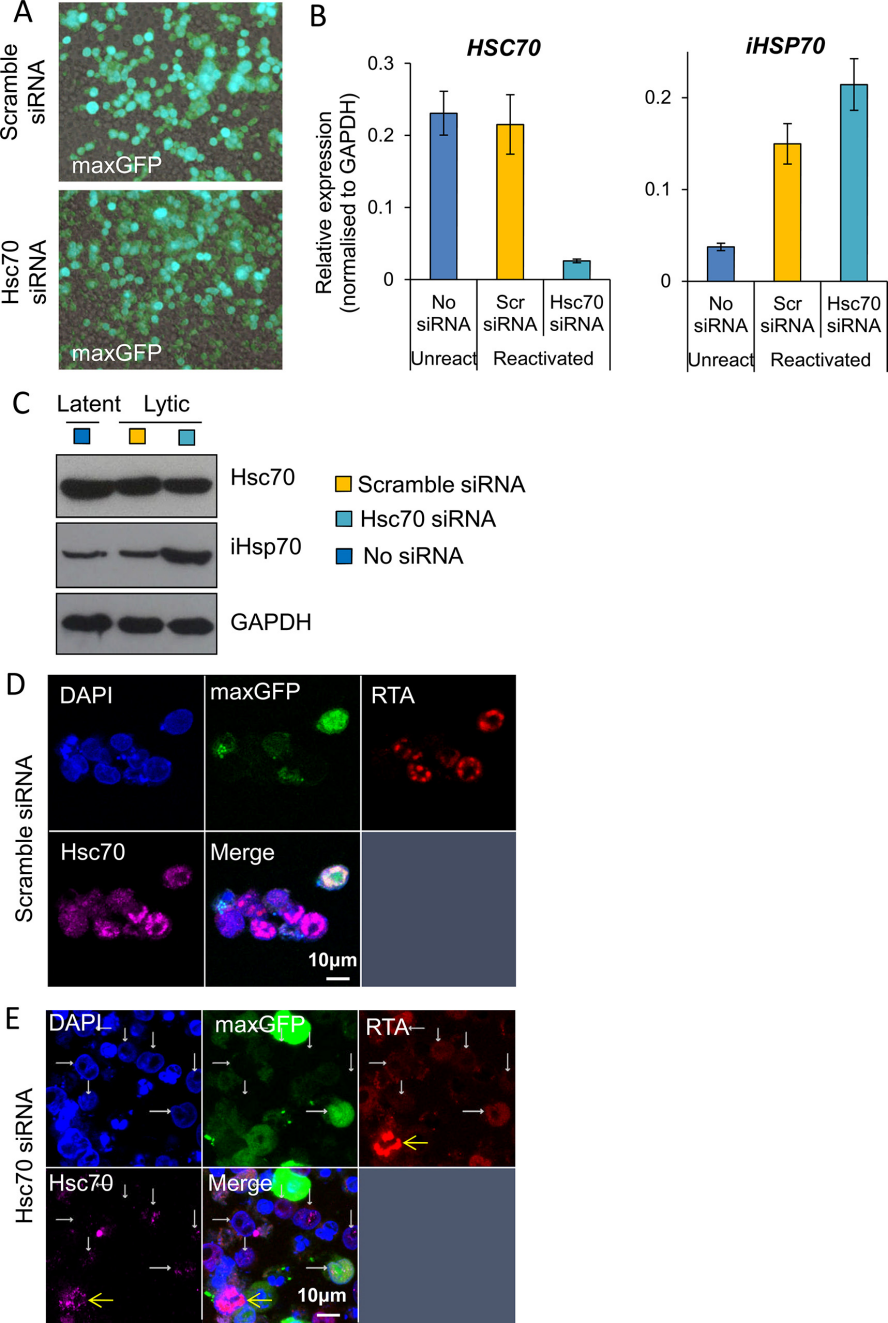
Depletion of Hsc70 abrogated KSHV RTC formation in TREx BCBL1-RTA cells. (A) Cells were nucleofected with either scramble or Hsc70-specific siRNA together with pmaxGFP to monitor transfection efficiency. 48 h post-nucleofection cells were imaged by fluorescent and light microscopy with a 40-times objective. Merge images are shown. (B) After four days post-nucleofection, total RNA was isolated and qRT-PCR carried out. *Hsc70* mRNA levels showed _~_ 90% knockdown, in contrast *iHsp70* mRNA was significantly increased. (C) A minor Hsc70 depletion at the protein level was observed at four days post-nucleofection while iHsp70 was clearly upregulated in Hsc70 siRNA-treated cells as shown by Western blotting. (D and E) Six days post-nucleofection cells were reactivated for 24 h and immunofluorescence was performed with RTA and Hsc70 specific antibodies. (D) In scramble siRNA-treated cells RTCs to which Hsc70 was recruited were observed. (E) In Hsc70 siRNA-treated cells, cells nearly or completely depleted of Hsc70 protein did not form RTCs (white arrows), while cells presenting assembled RTCs still displayed Hsc70 nuclear foci (yellow arrows).

To further support the essential role of Hsc70 during KSHV RTC formation, Hsc70 was specifically silenced in TREx BCBL1-RTA cells. For this, nucleofection was carried out. Transfection efficiency was monitored co-transfecting the Hsc70 siRNA together with pmaxGFP, which encodes maxGFP, a green fluorescent protein from the copepod *Pontellina p*. (**Fig 12A**). Note that higher transfection efficiency is expected for the siRNA due to the smaller size of this compared with the plasmid DNA. After four days post-nucleofection, *Hsc70* mRNA levels showed _~_ 90% knockdown (**Fig 12B**) with a minor depletion at the protein level (**Fig 12C**). Due to the stability of Hsc70 protein, cells were incubated for six days post-nucleofection followed by a further 24 h reactivation and immunofluorescence for Hsc70 and RTA was carried out. RTC formation dramatically decreased after nucleofection. Similar impairment in KSHV lytic replication has previously been reported in electroporated TREx BCBL1-RTA cells [74]; nevertheless, in scramble siRNA-treated cells, groups of cells could still be seen displaying RTCs to which Hsc70 was relocated (**Fig 12D**). In contrast, in Hsc70 siRNA-treated cells, fewer RTCs were visible and these exhibited nuclear Hsc70 (**Fig 12E yellow arrow**) while cells fully depleted of Hsc70, as identified by lack of Hsc70-labelling, did not form RTCs (**Fig 12D white arrows**). This result strongly suggests that Hsc70 is an essential chaperone for the formation of KSHV RTCs in TREx BCBL1-RTA cells.

## Discussion

Current quantitative proteomics approaches have become an invaluable tool for large-scale, high-throughput identification of proteins in complex biological samples. Moreover, advances in subcellular fractionation offer a way to further reduce the complexity of the samples to be analysed by LC-MS/MS, allowing identification of low abundance proteins. In this present study, we have developed a novel quantitative proteomic approach enhanced by subcellular fractionation that has enabled us to elucidate the cellular protein composition of KSHV RTCs. This novel approach led to the identification of several upregulated pathways in reactivated cells associated with the NE fraction (**S2 Table**). The first scored pathway was RNA post-transcriptional modification, the second highlighted pathway was protein synthesis with 26 different ribosomal proteins identified and the third scored pathway was DNA replication, recombination and repair. In addition, the isolation of the NE regions from unreactivated and reactivated cells followed by the uncommon use of urea for protein extraction led to the mass spectrometric identification of several Hsp70 isoforms and their respective co-chaperones at significant levels in NE-associated RTCs of reactivated cells. Immunoflourescence analysis confirmed that endogenous Hsc70 and iHsp70 were redistributed from the cytoplasm to the periphery of KSHV RTCs where they formed multiple nuclear foci during early lytic replication. The formation of RTCs coincided in time with the appearance of nuclear chaperone foci. Similar virus-induced-chaperone-enriched (VICE) domains that form adjacent to HSV-1 RTCs, have also been observed in HSV-1-infected cells and contain sequestered Hsc70, iHsp70, Hsp40 and Hsp90 [58, 75, 76]. HSV-1 induced-VICE domains also accumulate ubiquitinated proteins and components of the proteasome and function to sequester misfolded proteins away from RTCs and serve as protein quality control centers [75, 77]. iHsp70 redistribution very similar to that seen in HSV-1 induced-VICE domains was observed in multiple cells undergoing KSHV lytic replication, moreover this labelling was also observed for Hsc70 in some cells. Hsc70 and iHsp70 remain positioned exclusively adjacent to HSV-1 RTCs. However, in contrast; these chaperones were very dynamic during KSHV lytic replication and surprisingly very large chaperone foci were recruited within KSHV RTCs when viral DNA was actively synthesised (**Fig 2C and S1B Fig**). Therefore, our results strongly suggest that these chaperones may also aid replication of KSHV genomes during lytic replication. We therefore further suggest that Hsp70 isoforms may have an important role in the assembly and activation of pre-initiation complexes on the origin of DNA replication. This is supported by observations in prokaryotes, such as in plasmid P1 [78, 79], and eukaryotes, such as *Saccharomyces cerevisiae* [80], human papillomavirus-11 [81, 82], HSV-1 [83] and bacteriophage λ [84, 85]. Interestingly, Hsp70 isoforms have also been identified in the KSHV virion [86, 87] and therefore an additional role of these chaperones in KSHV capsid assembly may also be possible.

Cancer cells greatly rely on members of the *HSP70* and *HSP90* chaperone families for their growth and survival [5, 88], consequently, significant efforts have been invested to design small molecule inhibitors specific for these ATPases as novel anticancer therapeutics. This has been successfully achieved for the *HSP90* family with several inhibitors undergoing phase III clinical trials [14], although the clinical efficacy of these inhibitors has been somewhat limited because of the inevitably upregulation of iHsp70, when inhibiting Hsp90 [56, 57]. The development of *HSP70* inhibitors has substantially lagged behind that of *HSP90* inhibitors due to lack of natural product inhibitors specific for Hsp70 isoforms, due to the highly polar nature of the Hsp70/Hsc70 ATP binding site and the high affinity for ATP displayed by these ATPases [12]. Nevertheless, in recent years several *HSP70* inhibitors have been designed and tested in pre-clinical or clinical trials [88]. However, a major challenge remains in finding inhibitors which can specifically discriminate between iHsp70 and Hsc70. To date, only peptide aptamers targeting iHsp70 are selective for a specific Hsp70 isoform [89] and also recently, methyl blue was reported to specifically inhibit iHsp70 by oxidizing a cysteine in its ATPase domain, the same residue is absent in Hsc70 allowing differential targeting of the isoforms [90]. Because both Hsc70 and iHsp70 were specifically redistributed to KSHV RTCs during lytic replication, we made use of a recently developed small molecule inhibitor, VER-155008, which targets the ATPase pocket of the main three human Hsp70 isoforms. This inhibitor used at non-cytotoxic concentrations was able to effectively abrogate early and late KSHV transcription together with viral protein production, viral DNA replication and viral progeny.

Therefore, our study highlights the potential of VER-155008 or other novel *HSP70* inhibitors to prevent KSHV lytic replication in KSHV-associated tumours. One could expect that *HSP70* inhibition would be directly detrimental not only to cancer cell survival but also the virus-specific functions that are dependent on Hsp70 isoforms. These results may have exciting implications in combination with the recently demonstrated efficacy of ATP-competitive *HSP90* inhibitors in blocking KSHV latent cycle *in vitro* and in a xenograft KSHV tumour model [41]. It may be the case that combining *HSP70* inhibitors with *HSP90* inhibitors may lead to enhanced efficacy in eradicating latent KSHV reservoirs. Excitingly, in our cell culture models VER-155008 abrogated lytic replication without severely affecting cell viability or triggering apoptosis. It is intriguing that inhibition of constitutively expressed Hsc70 chaperone did not result in cell death. However, it is important to highlight previous studies carried out on the susceptibility of tumour cells versus normal cells to *HSP90* inhibitors. In tumour cells, Hsp90 is present entirely in multi-chaperone complexes with high ATPase activity; in contrast, in normal cells Hsp90 is in an uncomplexed conformation. These two distinct Hsp90 presentations result in tumour Hsp90 exhibiting a 100-fold higher binding affinity to an *HSP90* inhibitor than normal Hsp90 [91]. Thus, it is tempting to speculate that the multi-chaperone *HSP70* foci that are recruited to KSHV RTCs during lytic replication (**Fig 2 and S1 Fig**) are more sensitive to VER-155008 than the chaperones not assembled in VICE domains which carry out the housekeeping functions for cell survival. This would explain why *HSP70* inhibition had a profound effect on KSHV lytic replication without affecting cell viability and support the idea that isoform specificity may not be a requirement for treatment of KSHV-associated tumours.

To establish the essential role of Hsp70 isoforms during KSHV lytic replication, we examined several possible functions. It could be hypothesised that Hsp70 isoforms could be implicated in 1) stabilization of essential viral proteins, 2) clearing stalled RNAPII during times of robust viral transcription, 3) activation of viral promoters, 4) formation of KSHV RTCs. Initially we hypothesised that Hsp70 isoforms may be necessary for maintaining the stability of the key KSHV viral proteins RTA and/or ORF57. This was deemed a possibility as during KSHV latency, the essential viral latency associated nuclear antigen (LANA) protein has been recently shown to be a client protein of the Hsp90 chaperone and several *HSP90* inhibitors reduced the expression of LANA [41]. In addition, the lytic KSHV K1 glycoprotein has also been reported to be a client protein of Hsp90 [92]. However, inhibition of Hsp70 isoforms did not alter the half-life of RTA or ORF57 (**Figs 3D and 4D**). Alternatively, Hsc70 has been observed at the periphery of HSV-1 RTCs where it is also believed to aid in clearing stalled RNAPII from viral genomes during times of active transcription [76]. Indeed, the serine-2 phosphorylated form of RNAPII undergoes ubiquitination and robust proteasomal degradation during HSV-1 infection [93]. In contrast, in KSHV-reactivated cells, there was only a slight reduction of serine-2 phosphorylated RNAPII in comparison with unreactivated cells even at late times (24 h) post-reactivation (**Fig 3C**) and a significant decrease in the other RNAPII forms was not evident (**Fig 3C**). This suggests that although robust RNAPII degradation is a feature observed in virus infection, such as HSV-1 and influenza virus [55, 93], it may not be universally conserved. It is interesting to note that expression of a dominant-negative Hsc70 (K71M) that cannot hydrolyze ATP during HSV-1 infection resulted in prevention of serine-2 RNAPII degradation and RTCs formation [76]. However, inhibition of Hsp70 isoforms by VER-155008 did not prevent the slight degradation of phospho-serine-2 RNAPII protein (**Fig 3C**). Using transient transfections, we also demonstrated that Hsp70 isoforms were not directly involved in the activation of viral promoters (**Fig 7D and 7E**). Strikingly however, inhibition of Hsp70 isoforms precluded KSHV RTCs formation (**Fig 8**) and RNAPII re-localization to viral promoters (**Fig 9**), thus, blocking KSHV RTCs formation led to abolishment of global viral transcription and subsequent protein synthesis and viral DNA replication. These results, taken together with the differential Hsc70 and iHsp70 labelling seen in HSV-1 and KSHV-infected cells, suggest that these chaperones may be playing additional roles, such as participating in viral DNA replication and/or capsid assembly, in KSHV lytic infection compared with HSV-1 infection. Importantly, in both viruses, HSV-1 and KSHV, inhibition of Hsc70 ATPase function leads to a clear impediment in RTCs formation and presents a novel antiviral target for multiple herpesviruses. Moreover, as HSV-1 and KSHV belong to different subfamilies of the *Herpesviridae* family (α and γ subfamily respectively), the key role of Hsp70 isoforms in RTC formation may be conserved across all subfamilies. In support of this notion is the fact that Hsc70 and iHsp70 have also been reported to be incorporated in the virion of the β-herpesvirus HCMV [94] and the γ-herpesvirus EBV [95]. Our results also support that the finding of Hsc70 and iHsp70 chaperones in the KSHV virion [86, 87] a decade ago was not casual, and that these chaperones are essential for KSHV RTCs formation during lytic replication. iHsp70 has also been detected in the HSV-1 virion [96]. To support the conserved role of Hsp70 isoforms in herpesvirus infection, a recent study showed that cellular depletion of Hsc70 protein significantly reduced HSV-1 viral output in cell culture without adversely affecting cell viability. Depleting Hsc70 from the HSV-1 virion also significantly reduced viral production by more than 50% [97].

Hsp70 isoforms may be recruited to RTCs for several reasons. Firstly, the chaperone may sequester misfolded, modified or unwanted proteins away from RTCs. Alternatively, Hsp70 could produce a site for protein remodelling and/or degradation which may regulate or delay cellular pathways, such as the apoptosis cascade. Finally, it may aid subtlety to clear stalled RNAPII complexes during robust viral transcription and replication. An additional question which is yet to be addressed is the mechanism by which Hsp70 isoforms are recruited to RTCs. One intriguing possibility is observations made during HSV-1 infection. Here, HSV-1 ICP27 has been shown to interact with Hsc70 and is required for Hsc70 nuclear foci formation [76]. It will now be interesting to determine if the functional KSHV homologue ORF57, also interacts with Hsc70 and whether its nucleocytoplasmic shuttling ability is essential for Hsc70 nuclear import.

In summary, we have identified a new essential role for Hsp70 isoforms during the formation of RTCs in KSHV lytic replication. Importantly, our results suggest that *HSP70* inhibitors have the potential as novel KSHV antiviral agents and it would now be interesting to test these in conjunction with other molecular chaperone inhibitors, specifically *HSP90* inhibitors [41], which have the potential to eradicate latent KSHV reservoirs in both *in vitro* and *in vivo* tumour models.

## Materials and Methods

### Cell culture, antibodies and reagents

TREx-BCBL-1-RTA cells (kindly provided by Dr. Jae Jung, University of Southern California) are a BCBL-1-based, primary effusion lymphoma (PEL) B cell line that has been engineered to inducibly express exogenous Myc-tagged RTA by the addition of doxycycline, leading to a robust reactivation of the full KSHV lytic cycle [48]. The rKSHV.219 cell line (kindly provided by Dr. Jeffery Vieira, University of Washington, Seattle, USA) maintains KSHV as a latent infection and was generated by infecting HEK-293T cells (ATCC) with a recombinant KSHV that contains a constitutively active puromycin resistance and GFP gene, and an RFP gene that is fused to an RTA-responsive lytic cycle (PAN) promoter; hence, expression of RFP can be used as a reporter of RTA activity [42]. HEK-293T rKSHV.219 cells were grown in DMEM (Life Technologies) supplemented with 10% foetal calf serum (FCS) (Life Technologies) and 1% penicillin/streptomycin (P/S). This cell line was kept under puromycin (Sigma) selection (0.2 μg/ml). Reactivation into the lytic cycle was achieved by addition of 12-O-tetradecanoylphorbol 13-acetate (TPA) (20 ng/ml) and sodium n-butyrate (NaB) (Sigma) (4 mM). The TREx BCBL1-RTA cell line was grown in RPMI 1640 medium (Life Technologies) supplemented with 10% FCS and 1% P/S. This cell line was kept under hygromycin B (Life Technologies) selection (100 μg/ml) and inductions were performed using 2 μg/ml doxycycline hyclate (Sigma) as previously described [98]. All cells were maintained at 37°C in a humidified incubator with 5% CO_2_. Plasmid transfections were carried out using Lipofectamine 2000 (Life Technologies), as previously described [99]. Luciferase assay plasmids *Renilla* luciferase vector pRL-TK and firefly luciferase vector pGL3-BASIC were purchased from Promega, pEGFP-N1 was obtained from Clontech, pRTA-EGFP and pPAN-WT have been previously described [71, 100].

The monoclonal mouse antibodies to anti-nuclear pore complex proteins (mAb414) (ab24609), GAPDH (6C5), rabbit polyclonal anti-lamin B1 and anti-Ser2 RNAPII were purchased from Abcam. The rabbit polyclonal anti-histone H3 C-terminus (39164) was purchased from Active Motif. The rabbit polyclonal anti-Nup160 was obtained from Bethyl Laboratories. Monoclonal antibodies to KSHV ORF57 (207.6), to Hsc70 (B-6), to Grp78 (A-10), to Hsp90 (4F10), to B-23 (0412) and to C-23 (H6) were obtained from Santa Cruz. The mouse monoclonal (C92F3A-5) anti-iHsp70 was from Enzo Life Sciences. The rabbit polyclonal anti-PARP1 was purchased from Cell Signalling. The mouse monoclonal (CTD4H8) anti-RNAPII was purchased from Millipore. The mouse monoclonal (9E10) anti-c-Myc was from Sigma. Sheep anti-KSHV minor capsid protein was purchased from Exalpha Biologicals, Inc. The rabbit polyclonal anti-RTA was a gift from Professor David Blackbourn (University of Surrey, UK). The mouse monoclonal (JL-8) anti-GFP was supplied by Clontech. The inhibitor for Hsp70 isoforms (VER-155008) was obtained from Tocris Bioscience.

### SILAC and nuclear envelope isolation

For SILAC, HEK-293T cells rKSHV.219 were fed with either medium (R6K4) or light (R0K0) labelled medium (Dundee Cell Products) containing 10% dialysed FCS (Dundee Cell Products) for six passages to allow incorporation of the isotopes, as previously described [101]. Subsequently, to induce lytic replication three T175 flasks were reactivated with TPA (20 ng/ml) and NaB (4mM) for 48 h, while another three T175 flasks remained unreactivated as control. To isolate NEs a protocol published by Korfali et al., [35] was used with minor modifications. 75 million cells were used per experimental condition. Cells were washed with PBS and incubated in hypotonic lysis buffer (10 mM HEPES pH 7.4, 1.5 mM MgCl_2_, and 10 mM KCl) for 30 min followed by homogenization with a tight Dounce homogenizer. To stabilise and avoid lysing the nuclei after the hypotonic swelling step, cells were resuspended in 2.2M SHKM (2.2 M sucrose, 50 mM HEPES pH 7.4, 25 mM KCl, and 5 mM MgCl_2_) and 1M KCl. The resuspended cells were then underlayed with 30% SHKM (0.9 M sucrose, 50 mM HEPES pH 7.4, 25 mM KCl, and 5 mM MgCl_2_) and nuclei were pelleted at 2,000x*g* for 20 min at 4°C in a Eppendorf centrifuge 5804 R. Nuclei were resuspended in 1.9 M SHKM, underlayed with 2.2 M SHKM and transferred to 38.5-ml ultracentrifuge tubes (Beckman Coulter). Nuclei were then centrifuged at 82,000x*g* for 2h at 4°C in a Sorvall Discovery 90SE ultracentrifuge. Pellets were resuspended in 0.25 M SHKM (50 mM HEPES pH 7.4, 25 mM KCl, and 5 mM MgCl_2_), treated with 1% Triton-X in 10% SHM (0.3 M sucrose, 10 mM HEPES, pH 7.4, 2 mM MgCl_2_ and 0.5 mM Ca Cl_2_) for 10 min and centrifuged at 2,000x*g* for 10 min. Nuclei were then treated with RNase A (Thermo Scientific) and DNase I (Life Technologies) for 15 min, pelleted at 6,000x*g* for 10 min, resuspended in 10% SHM and treated again with RNase A and DNase I for 15 min. Nuclei were centrifuged at 2,000x*g* for 10 min, resuspended and incubated for 15 min in 10% SHM containing 0.3 M NaCl to remove nucleoplasmic contents. Nuclear envelopes were then pelleted at 1,500x*g* for 15 min. Insoluble nuclear envelope proteins were solubilised for 10 min in PBS supplemented with 0.1% Triton-X100 and 6 M urea. Samples were centrifuged at 6,000 *g* for 2 min to remove insoluble material and the supernatant containing nuclear envelope proteins was stored at -80°C for further Western blotting and mass spectrometry analysis. All solutions had freshly added 1x Complete, EDTA-free protease inhibitors (Roche). DTT (2 mM) was also freshly added to the solutions specified on Korfali’s protocol. LC-MS/MS was performed as previously described [22]. Bioinformatical analysis was performed with the Ingenuity Systems software packet, IPA 9.0 (Ingenuity Systems, Inc).

### Immunoprecipitation and Western blotting

Protein samples were extracted using lysis buffer containing 50 mM Tris (pH 7.4), 150 mM NaCl, 1% NP-40 and 1x Complete, EDTA-free protease inhibitors (Roche) for 30 min on ice, as previously described [102]. Protein samples were run on SDS-PAGE gels and transferred to nitrocellulose membranes (Amersham) via wet transfer. Membranes were blocked with TBS + 0.1% Tween 20 and 5% dried skimmed milk powder. Membranes were probed with relevant primary and secondary antibodies, treated with EZ-ECL (Geneflow) and exposed to Amersham hyperfilm ECL (GE Healthcare). Secondary antibodies were horseradish peroxidase (HRP)-conjugated polyclonal goat anti-mouse and polyclonal goat anti-rabbit (Dako). HRP-conjugated polyclonal rabbit anti-sheep was from Santa Cruz. GFP-Trap (Chromotek) experiments were performed as previously described [103]. Nuclear/cytoplasmic fractionations were performed as previously described [44], with the exception that nuclear pellets were solubilised for 20 min in PBS supplemented with 0.1% Triton-X100 and 6 M urea. Vigorous pipetting and vortexing was applied to the nuclear pellet. After urea treatment, insoluble material was removed by centrifugation at 6,000 *g* for 2 min and the supernatant kept for further analysis.

### Immunoflourescence

Cells were cultured overnight on poly-L-lysine (Life Technologies) coated glass coverslips in 24-well plates. Cells were fixed with 4% formaldehyde (Calbiochem) for 10 min and permeabilised with 0.1% Triton X-100 for 20 min as previously described [104]. For labelling with Grp78 antibody cells were fixed with ice-cold 100% methanol for five min. After permeabilization, cells were then incubated in blocking solution (PBS with 1% BSA) for 1 h at 37°C. Primary antibodies anti-Hsc70 (diluted 1:200), anti-iHsp70 (1:50), anti-Grp78 (1:50), anti-RNAPII (CTD4H8) (1:500) or rabbit RTA (1:1,000) were incubated for 1 h at 37°C. Coverslips were washed five times with PBS, incubated with appropriate secondary antibody for 1 h at 37°C, washed five times with PBS again and mounted in VECTASHIELD with DAPI (Vector Labs). Images were obtained using a LSM 510 META confocal microscope (Carl Zeiss) and processed using ZEN 2009 imaging software (Carl Zeiss) as previously described [105]. Fluorescently-conjugated secondary antibodies were all obtained from Life Technologies: Alexa Flour 633 goat anti-mouse IgG, Alexa Flour 488 goat anti-mouse IgG, Alexa Flour 488 goat anti-rabbit IgG, Alexa Flour 546 donkey anti-mouse IgG and Alexa Flour 546 goat anti-rabbit IgG.

### Edu assay

TREx BCBL1-RTA cells were labelled using the Click-iT EdU Alexa Fluor 647 Imaging Kit (Life Technologies) according to the manufacturer’s instructions with minor modifications as follows. Cells were seeded onto poly-L-lysine treated coverslips in 24-well plates followed by induction and incubation at 37°C for 24 hours. Prior to cell fixation, 10 μM EdU (5-ethynyl-2’-deoxyuridine) was added to each well for 45 min. Cells were then fixed for 10 min in 4% formaldehyde and permeabilised in 1% Triton X-100 for 20 min. Edu detection was carried out adding the Click-iT reaction cocktail for 30 min and immunofluorescent labelling for RTA and Hsc70 was performed as above. Cells were mounted in VECTASHIELD with DAPI (Vector Labs).

### Two-step quantitative reverse transcription PCR (qRT-PCR)

Total RNA from cells was extracted using TRIzol (Life Technologies) according to the supplier’s protocol. DNA-*free* DNA Removal Kit (Ambion) was used to remove any contaminating DNA from RNA samples. Reverse transcription was performed with ProtoScript II (NEB) and oligo(dT) primers and 1.5 μg of total RNA. Negative control reactions were performed in the same manner but without reverse transcriptase. Quantitative PCR (qPCR) reactions (20 μl) included 1X SensiMix SYBR green master mix (Bioline), 0.5 μM of each primer and 5 μl template cDNA (used at 1:200 dilution in RNase-free water). Cycling was performed in a RotorGene Q machine (Eppendorf). The cycling programme was a 10 min initial preincubation at 95°C, followed by 40 cycles of 95°C for 15 sec, 60°C for 30 sec and 72°C for 20 sec. After qPCR, a melting curve analysis was performed between 65 and 95°C (with 0.2°C increments) to confirm amplification of a single product. Relative expression compared to control cells was calculated using the ΔΔC_*T*_ method as previously described [106]. For each gene of interest and housekeeping gene (*GAPDH)* a standard curve was constructed using a pool of cDNA derived from unreactivated and reactivated cells. Six different dilutions of the standards were quantified, these included 1:100, 1:200, 1:400, 1:800, 1:1,600 and 1:3,200 dilution. The slope of the standard curve was used to calculate the amplification efficiency (AE) of the primers using the formula: AE = (10^−1/slope^). The mean cycle threshold (C_*T*_) was determined from three independent biological replicates. All genes of interest were normalised against the housekeeping gene *GAPDH* (ΔC_*T*_). ΔΔC_*T*_ was calculated subtracting ΔC_*T*_ of unreactivated cells from ΔC_*T*_ of reactivated cells and the fold change was then determined using AE ^(-ΔΔC^_*T*_^)^. Statistical significance was validated by Student’s *t*-test.

### Viral DNA replication assays

Unreactivated TREx BCBL1-RTA cells treated with DMSO (0.1%) were used as control to assess viral reactivation. Reactivated cells were exposed to doxycycline for 72 h. Total DNA was then isolated with the use of a QIAamp DNA mini kit (Qiagen) as per the manufacturer’s instructions. qPCR was carried out as described above. 10 ng of template DNA and primers specific for the *ORF57* gene were used. Quantification of *GAPDH* gene was used to normalize between samples and the mean cycle threshold (C_*T*_) was determined from three independent biological replicates. Relative levels of viral DNA compared with unreactivated cells were calculated using the ΔΔC_*T*_ method as previously described [106].

### Viral re-infection assays

TREx BCBL1-RTA cells that had been seeded on 12-well plates were reactivated and treated with control DMSO (0.1%) or VER-155008. Unreactivated cells treated with DMSO (0.1%) were used as control to evaluate viral reactivation. After 72 h reactivation, 700 μl of the RPMI 1640 culture medium was centrifuged at 800 *g* for five min, immediately mixed with 300 μl of DMEM supplemented with 10% FCS and 1% P/S and incubated for a further 24 h with HEK-293T cells that had been seeded in 12-well plates the previous day. Total RNA was then extracted with TRIzol (Life Technologies) and qRT-PCR carried out as described above. Relative expression compared to control cells was calculated using the ΔΔC_*T*_ method as previously described [106].

### Proliferation (MTS) assay

Determination of the cellular metabolic activity was performed using a non-radioactive CellTiter 96 AQ_ueous_ One Solution Cell Proliferation Assay (MTS) (Promega), according to the manufacturer's manual. 20,000 cells TREx BCBL1-RTA or 10,000 HEK-293T rKSHV.219 cells were seeded in triplicate in a flat 96-well culture plate (Corning). After 24 h inhibitor exposure, CellTiter 96 AQueous One Solution Reagent was added and cells were incubated for 1 h in a humidified incubator in 5% CO_2_ at 37 °C. Absorbance was measured at 490 nm using an Infinite F50 (Tecan) plate reader. Background control had culture medium without cells and the signal from this was subtracted to all other absorbance values.

### ApoTox-Glo Triplex Assay

This assay allows evaluation of viability, cytotoxicity and effector caspases activation within a single assay well. The assay was carried out as specified on the supplier’s manual. 20,000 TREx BCBL1-RTA cells or 10,000 HEK-293T rKSHV.219 cells were seeded in triplicate in tissue culture treated black microplates (Greiner Bio-One). No-cell control (background) contained only culture medium and the signal from this was subtracted to all other absorbance and luminescence values. Fluorescence and luminescence readings were collected using a GloMax System (Promega) (kindly provided by Dr. John Boyle, University of Leeds, UK).

### Dual-Luciferase assay

Luciferase activity was detected using the Dual-Luciferase Reporter Assay System (Promega) as previously described [107]. HEK-293T cells were seeded in triplicate in flat 96-well culture plate (Corning) at a density of 10,000 cells per well. Following the respective plasmid transfections and inhibitor exposure, media was removed from the culture wells and cells washed gently with 100 μl PBS. 30 μl 1x passive lysis buffer was added to the cell monolayer which was rocked for 15 min and then 20 μl of each lysate was transferred to tissue culture treated white microplates (Greiner Bio-One). Luciferase measurements were carried out in a FLUOstar Optima microplate reader (BMG Labtech Ltd), with injectors 1 and 2 being used to dispense 50 μl of Luciferase Assay Reagent II and Stop & Glo Reagent respectively. Firefly luciferase activity was normalized to *Renilla* luciferase activity.

### Chromatin immunoprecipitation (ChIP)

Formaldehyde-crosslinked chromatin was prepared using the Pierce Chromatin Prep Module (Thermo Scientific) following the manufacturer’s protocol. 2 x 10^6^ cells were used per experimental sample and digested with six units of micrococcal nuclease (MNase) per 100 μl of MNase Digestion buffer in a 37°C water bath for 15 min. These conditions resulted in optimal sheared chromatin with most chromatin fragments ranging from 150–300 base pairs. Immunoprecipitations were carried out using EZ-ChIP kit (Millipore) according to the supplier’s instructions and as previously described [44]. Immunoprecipitations were done overnight at 4°C and contained 50 μl of digested chromatin (2 x 10^6^ cells), 450 μl of ChIP dilution buffer and 1.5 μg of RNAPII antibody (clone CTD4H8) (Millipore) or isotype antibody, normal mouse IgG (Millipore). Both antibodies were provided with the EZ-ChIP kit. Prior to qPCR analysis, eluted DNA was subjected to a DNA clean up step using UltraClean PCR Clean-Up Kit (Mo Bio Laboratories) according to the supplied protocol with the exception of using 500 μl of SpinClean buffer instead of 300 μl. qPCR reactions were performed as described above and using either 2 μl of ChIP’ed DNA or 2 μl of input DNA as template.

### siRNA Knockdown

HEK-293T rKSHV.219 cells seeded on 12-well plates were reverse transfected with either 100 nM of the specific Silencer Select siRNA (Life Technologies) or 100 nM AllStars negative control siRNA (Qiagen) using 7 μl of siPORT NeoFX transfection agent (Life Technologies) per transfection. The siRNA ID for *Hsc70* and *iHsp70* were s6985 and s6968 respectively. s6968 siRNA targets the two major iHsp70 proteins (HSP70-1 and HSP70-2). Two days post-transfection, cells were transfected again in the same manner. Four days after the first transfection, cells were reactivated and incubated for the desired time. Proteins and total RNA were isolated with TRIzol (Life Technologies) and subsequent Western blot and qRT-PCR were performed.

8 × 10^6^ TREx BCBL1-RTA cells were transfected once with 100 μl of Nucleofector solution V (Lonza) to which 2 μM siRNA (scramble or Hsc70) was added. In addition, to monitor transfection efficiency, 1 μg of the control plasmid pmaxGFP was also co-transfected. Cells were transfected using program T-01 of an Amaxa nucleofector I (Lonza). After nucleofection cells were maintained in six-well plates. Medium was freshly replaced every day.

### Confocal profiling analysis

Confocal images were subjected to profiling analysis using Zeiss Zen 2011 software. This involved drawing a line in a confocal image to measure the relative intensity of each channel at every pixel along the line. Profiling was conducted for each cell in two representative confocal images taken with a 40-times objective. These data were then analysed using Microsoft Excel 2010. Firstly, a function was used to define whether the relative intensity of a pixel could be defined as a “peak” and thus an Hsc70 foci. This function asked whether the data point for one specific pixel of the rhodamine channel was ≥ 20, this was set as the arbitrary threshold to eliminate background noise. If this condition was met, the function then asked whether the data point was greater than or equal to the data point in the previous and subsequent pixel. If these conditions were true, then this data point was counted as a peak. This was performed for every data point measured in the line profile providing the total number of Hsc70 peaks in the profile of one cell. Next, another function was used to determine whether the relative intensity in the DAPI channel was ≥ 25, a threshold determined from visualising the line profiling data as a graph. If a pixel was shown to exceed the threshold in the DAPI channel and also in the rhodamine channel, then it was counted as a nuclear Hsc70 peak. These measurements were conducted for each pixel in each profile allowing counting Hsc70 nuclear peaks in each profile. Hsc70 peaks outside the nucleus corresponded to cytoplasmic Hsc70 peaks.

### Oligonucleotides

Oligonucleotide primer sequences are available upon request. All primers were purchased from Sigma (UK).

## Supporting Information

S1 Table Cellular proteins previously reported to localise to herpesvirus RTCs and found significantly increased in the NE of reactivated cells. (PDF)

S2 Table Bioinformatic analysis of the cellular proteins and their associated canonical pathways upregulated in nuclear envelopes during KSHV infection. (PDF)

S1 Dataset List of all proteins identified by LC-MS/MS in the NE-fractions of unreactivated and reactivated HEK-293T rKSHV.219 cells. (XLS)

S1 Fig iHsp70 was redistributed from the cytoplasm to both the periphery and within KSHV-induced RTCs. (A) TREx BCBL1-RTA cells remained unreactivated or reactivated for either 20 h or 24 h. In unreactivated cells iHsp70 was cytoplasmic (i). In contrast, at 20 h reactivation an increase in nuclear iHsp70 labelling was seen with numerous small iHsp70 foci found mainly adjacent to viral RTCs (ii). Some cells displayed iHsp70 completely recruited within RTCs (iii and iv asterisks), while other cells accumulated large iHsp70 adjacent to RTCs (iv arrows). (B) TREx BCBL1-RTA cells remained unreactivated (i) or reactivated for 24 h (ii and iii) followed by triple-labelling with antibodies specific for RTA and iHsp70 and Click-iT EdU Alexa Fluor 647. Complete co-localisation between iHsp70, RTA and actively replicated viral DNA (Edu-labelled) was observed in both incipient RTCs (ii) and in fully-developed RTCs (iii). Note that in these cells iHsp70 was not depleted from the cytoplasm.
(TIF)

S2 Fig Grp78 was not redistributed to KSHV RTCs during lytic replication in TREx BCBL1-RTA cells. This finding is consistent with the ER retention signal found in Grp78.
(TIF)

S3 Fig iHsp70 and Hsc70 formed nuclear foci in HEK-293T rKSHV.219 cells undergoing lytic replication while Grp78 was not redistributed to RTCs. (A and B) Cells undergoing lytic replication as identified by red fluorescent protein (RFP) expression displayed iHsp70 and Hsc70 nuclear foci that appeared to assemble in RTCs. (C) The endoplasmic reticulum (ER) Hsp70 isoform, named Grp78, remained in the ER regardless of lytic reactivation.
(TIF)

S4 Fig Proliferation (MTS) assay in unreactivated TREx BCBL1-RTA cells exposed to VER-155008 for 24 h. Cell metabolic activity was drastically reduced at 6.25 μM VER-155008.
(TIF)

S5 Fig EGFP-RTA expression redistributed endogenous iHsp70 from the cytoplasm to the nucleus. HEK-293T cells were transfected with control pEGFP or pRTA-EGFP for 24 h and then analysed by immunofluorescence.
(TIF)

S6 Fig ApoTox-Glo Triplex Assay in HEK-293T cells revealed that VER-155008 did not increase cytotoxicity nor activate effector caspases. Cytotoxicity of VER-155008 was assessed in cells exposed to increasing inhibitor concentrations for 24 h. Even at 60 μM VER-155008 there was no caspase 3/7 activation compared with DMSO control cells.
(TIF)

S7 Fig Nuclear Hsc70 co-localised with viral DNA in KSHV RTCs. TREx BCBL1-RTA cells were reactivated for 24 h in the presence of control DMSO (0.1%) or 2 μM VER-155008 followed by labelling with Click-iT EdU Alexa Fluor 647 and an antibody specific for Hsc70. (A) In DMSO-treated reactivated cells, Hsc70 formed multiple nuclear foci. Three cells showing viral RTCs filled with viral DNA (Edu-labelled) which co-localised with Hsc70 foci can be seen. (B) Cells treated with VER-155008 displayed Hsc70 protein distributed more equally between the nucleus and cytoplasm and RTCs replicating viral DNA were not as abundant as in DMSO-treated cells.
(TIF)

S8 Fig Higher magnification of Fig 9Bii. VER-155008 at 2 μM abrogated RNAPII recruitment to KSHV RTCs.
(TIF)

S9 Fig VER-155008 at 2 μM abrogated RNAPII recruitment to KSHV RTCs. TREx BCBL1-RTA cells remained unreactivated or reactivated for 24 h in the presence of control DMSO (0.1%) or 2 μM VER-155008 followed by labelling with Click-iT EdU Alexa Fluor 647 and an antibody specific for RNAPII (clone CTD4H8). (A) A high proportion of unreactivated TREx BCBL1-RTA cells replicated their cellular DNA (Edu-labelled) in the presence of control DMSO (0.1%) or 2 μM VER-155008. Normal RNAPII localization was observed in these cells, with nuclear RNAPII excluding the nucleoli. (B) In contrast, reactivated cells entered cell cycle arrest as demonstrated by fewer Edu-labelled cells. In the presence of DMSO, multiple RTCs were formed with some replicating viral DNA (white arrows). In cells treated with VER-155008, multiple pre-replicative sites were seen labelled by RNAPII antibody and Edu-labelling was more diffused in the nucleus compared with DMSO-treated cells.
(TIF)
