# Supplementary material for: Hsp70 Isoforms Are Essential for the Formation of Kaposi’s Sarcoma-Associated Herpesvirus Replication and Transcription Compartments
Source: PLoS Pathog. 2015 Nov 20;11(11):e1005274. doi: 10.1371/journal.ppat.1005274 (PMC4654589; doi:10.1371/journal.ppat.1005274)
Supplement: S1 Table — (PDF) [file ppat.1005274.s001.pdf]

Gene	Uniprot number	Ratio Reactivated/ Unreactivated	Number of unique peptides
CSNK2A1	E7EU96	3.091	14
CSNK2B	P67870	2.812	6
CSNK2A2	P19784	2.477	6
BLM	H0YNU5	12.941	6
TOP2A (topoisomerase IIα)	P11388	2.399	52
DDX5	B4DLW8	2.622	47
DDX17	Q92841	2.172	54
RPL11	Q08ES8	2.471	16
HIST1H1C	P16403	2.271	31
HNRNPA0	Q13151	2.393	9
HNRNPH1	E9PCY7	1.964	9
HNRNPF	P52597	1.852	3
RBBP7	Q5JNZ9	1.805	2
TOP1 (topoisomerase I)	P11387	1.856	10
HDAC2	B3KRS5	1.853	7
PCNA	P12004	1.633	3
DDX21	Q9NR30	1.615	76
RCOR1	Q9UKL0	1.662	5
EIF6	P56537	1.515	18
HNRNPA1	F8VXY0	1.620	30
HNRNPA2B1	P22626	1.489	49
HNRNPM	P52272	1.427	101
