# Supplementary material for: Hsp70 Isoforms Are Essential for the Formation of Kaposi’s Sarcoma-Associated Herpesvirus Replication and Transcription Compartments
Source: PLoS Pathog. 2015 Nov 20;11(11):e1005274. doi: 10.1371/journal.ppat.1005274 (PMC4654589; doi:10.1371/journal.ppat.1005274)
Supplement: S2 Table — (PDF) [file ppat.1005274.s002.pdf]

Gene	UniProt number	Ratio Reactivated/ Unreactivated	Number of unique peptides
1. RNA Post-Transcriptional modification, Cardiovascular disease, Cellular assembly and organisation			
AASDHPPT	Q9NRN7	1.948	4
AKAP8L	Q9ULX6	2.036	4
ALYREF	Q86V81	3.615	3
CHTOP	Q9Y3Y2	2.017	21
CPSF6	F8WJN3	2.212	3
CSNK2A1	E7EU96	3.091	14
CUL3	Q53S54	1.959	6
DAP3	P51398	1.930	3
DDX5	B4DLW8	2.622	47
DDX17	Q92841	2.172	54
DDX24	G3V529	2.371	16
DHX8	B7Z8F4	2.248	10
HNRNPH1	E9PCY7	1.964	9
KHSRP	Q92945	2.254	4
SRSF2	J3QL05	2.042	15
MRPS7	J3QKW2	4.038	3
NLE1	Q9NVX2	2.017	8
NONO	F5GYZ3	2.044	26
PSPC1	B4DWI8	5.723	4
SFPQ	Q9BSV4	2.041	20
RBM7	J3KPD3	3.052	3
RBM14	Q96PK6	2.411	46
RBM15	A1A693	2.289	15
RBMX	P38159	2.150	25
RNF40	H3BP71	1.968	12
SAP30BP	Q9UHR5	1.991	9
SRRT	Q9BXP5	1.917	12
TMPO	P42166	2.761	6
MAGED2	Q5H909	3.312	4
PDIA6	B7Z254	2.553	4
WDR83	Q9BRX9	13.246	3
TRAP-1	Q12931	2.003	9
SP1	B4DJU4	2.608	7
2. Gene expression, Protein synthesis, RNA post-transcriptional modification			
CTBP2	P56545	2.067	3
EIF2S3	Q53HK3	2.651	3
IMPDH2	H0Y4R1	2.218	6
RPL8	P62917	3.018	12
RPL11	Q08ES8	2.471	16
RPL13	P26373	1.960	12
RPL14	A8K7N0	2.283	14
RPL15	E7EQV9	1.951	7

RPL18	F8VWC5	2.042	25
RPL19	J3QR09	2.083	7
RPL21	G3V1B3	2.405	7
RPL24	C9JXB8	2.138	8
RPL27	E4W6B6	2.151	7
RPL30	E5RI99	2.400	10
RPL31	H7C2W9	3.705	3
RPL36	Q9Y3U8	2.041	4
RPL18A	B4DM74	14.807	6
RPL23A	P62750	2.012	18
RPL35A	F8WBS5	2.815	3
RPL7A	P62424	2.400	15
RPS2	H0YEN5	2.454	8
RPS3	P23396	2.369	22
RPS11	P62280	2.366	7
RPS14	P62263	2.400	14
RPS16	P62249	3.216	13
RPS27	P42677	2.115	5
RPS3A	P61247	1.947	19
RPS4X	Q96IR1	2.821	20
RRP36	Q96EU6	2.880	14
TSR1	Q2NL82	2.545	5
3. DNA replication, recombination and repair, Cell cycle, cellular assembly and organization			
ATAD5	Q96QE3	2.115	5
BLM	H0YNU5	12.941	6
CENPF	P49454	1.948	39
DDX3X	B5BTY4	1.961	25
EIF3A	Q05BS0	2.316	3
EIF3E	B3KW56	2.210	3
EIF3F	B4DMT5	2.698	6
EIF3I	Q53HU7	4.908	4
EIF4A1	A8K7F6	3.468	9
FEN1	P39748	2.365	4
KIF20B	Q96Q89	2.669	10
KIF4A	O95239	2.323	10
KIFC1	B4E063	3.063	13
NCAPD2	E7EN77	2.403	3
NCAPD3	P42695	4.007	3
NCAPG	Q6NUR1	2.610	4
NSF	B7Z5J7	2.773	6
PLS3	Q96HI1	2.251	3
PRC1	F8W9B5	2.679	4
RAB5C	P51148	2.354	4
RFC2	P35250	2.056	17
RFC3	P40938	1.948	19

RFC5	A8K3S0	2.129	15
RPS10	P46783	5.334	7
RPS18	P62269	3.042	17
SMC	B3KMB1	2.446	12
WRNIP1	Q96S55	6.319	5
