# Supplementary figures and images for: Hsp70 Isoforms Are Essential for the Formation of Kaposi’s Sarcoma-Associated Herpesvirus Replication and Transcription Compartments
Source: PLoS Pathog. 2015 Nov 20;11(11):e1005274. doi: 10.1371/journal.ppat.1005274 (PMC4654589; doi:10.1371/journal.ppat.1005274)

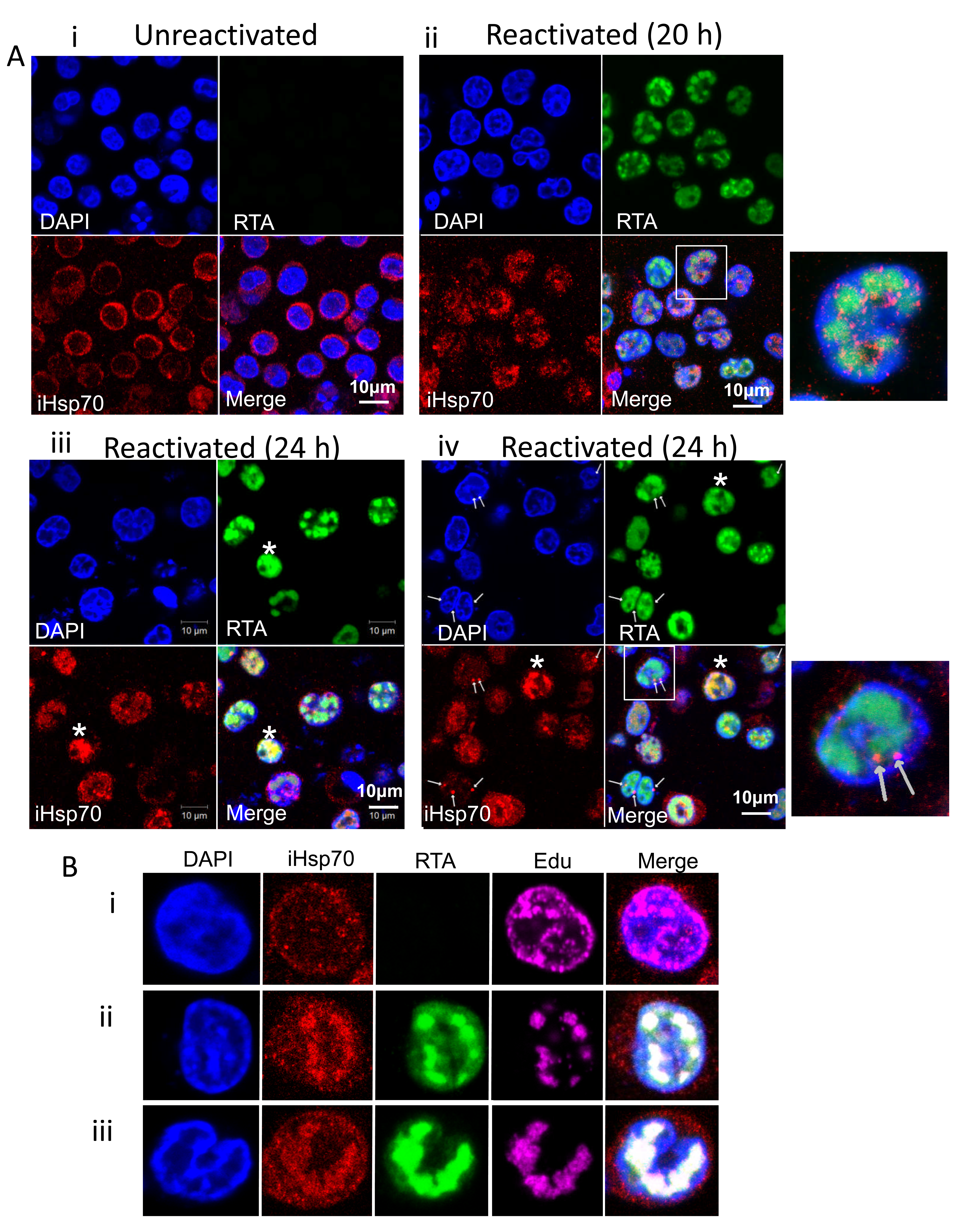

Supplement: S1 Fig — (A) TREx BCBL1-RTA cells remained unreactivated or reactivated for either 20 h or 24 h. In unreactivated cells iHsp70 was cytoplasmic (i). In contrast, at 20 h reactivation an increase in nuclear iHsp70 labelling was seen with numerous small iHsp70 foci found mainly adjacent to viral RTCs (ii). Some cells displayed iHsp70 completely recruited within RTCs (iii and iv asterisks), while other cells accumulated large iHsp70 adjacent to RTCs (iv arrows). (B) TREx BCBL1-RTA cells remained unreactivated (i) or reactivated for 24 h (ii and iii) followed by triple-labelling with antibodies specific for RTA and iHsp70 and Click-iT EdU Alexa Fluor 647. Complete co-localisation between iHsp70, RTA and actively replicated viral DNA (Edu-labelled) was observed in both incipient RTCs (ii) and in fully-developed RTCs (iii). Note that in these cells iHsp70 was not depleted from the cytoplasm. (TIF) [file ppat.1005274.s004.tif]

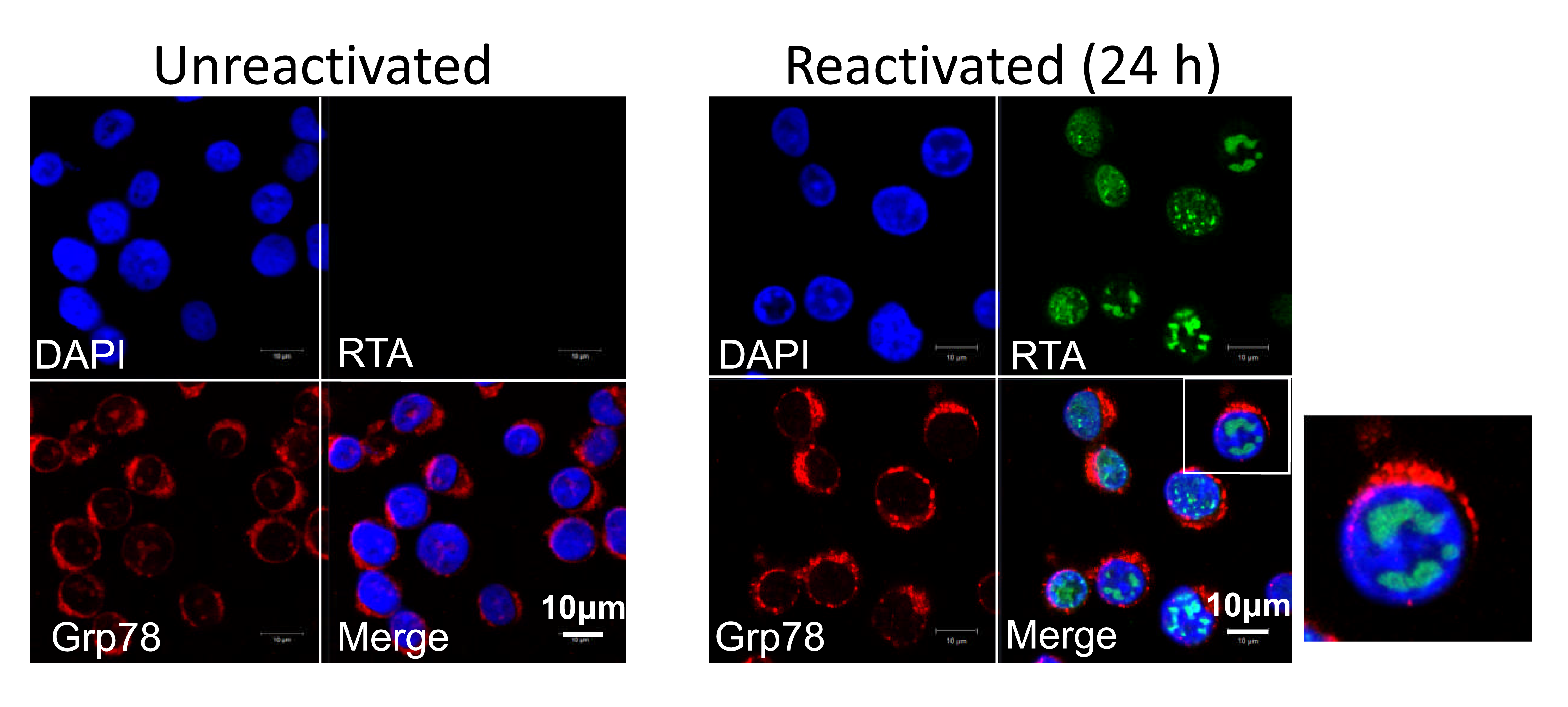

Supplement: S2 Fig — This finding is consistent with the ER retention signal found in Grp78. (TIF) [file ppat.1005274.s005.tif]

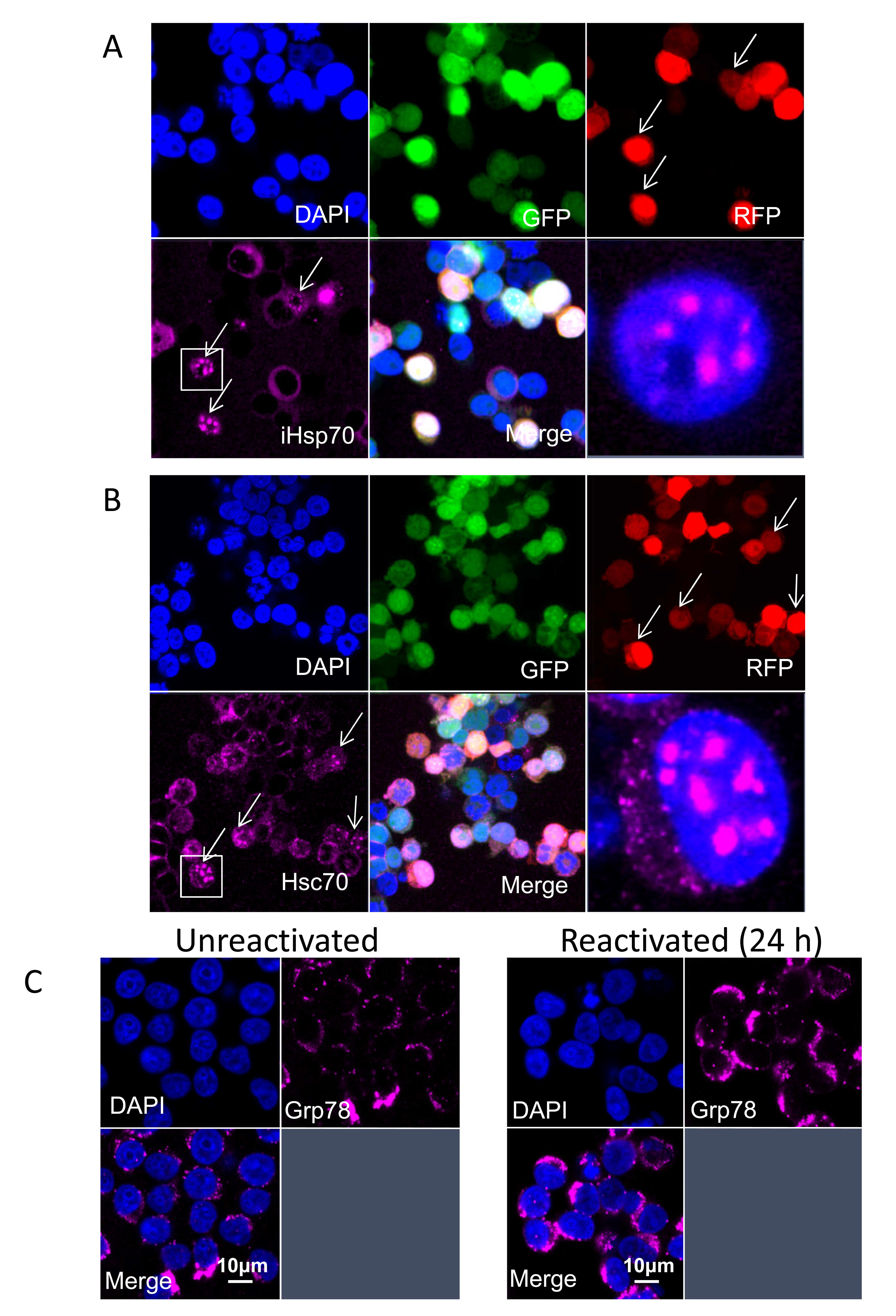

Supplement: S3 Fig — (A and B) Cells undergoing lytic replication as identified by red fluorescent protein (RFP) expression displayed iHsp70 and Hsc70 nuclear foci that appeared to assemble in RTCs. (C) The endoplasmic reticulum (ER) Hsp70 isoform, named Grp78, remained in the ER regardless of lytic reactivation. (TIF) [file ppat.1005274.s006.tif]

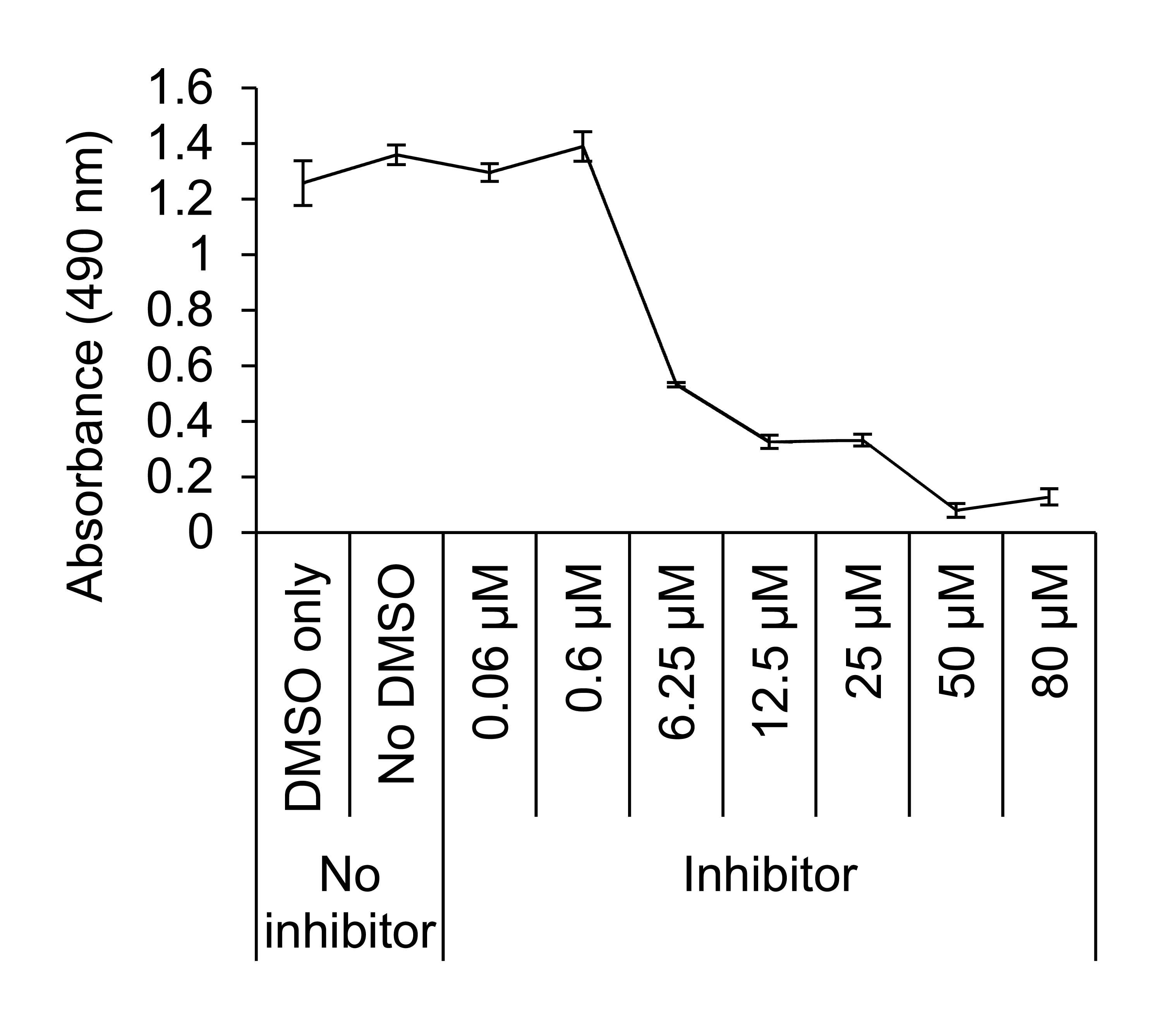

Supplement: S4 Fig — Cell metabolic activity was drastically reduced at 6.25 μM VER-155008. (TIF) [file ppat.1005274.s007.tif]

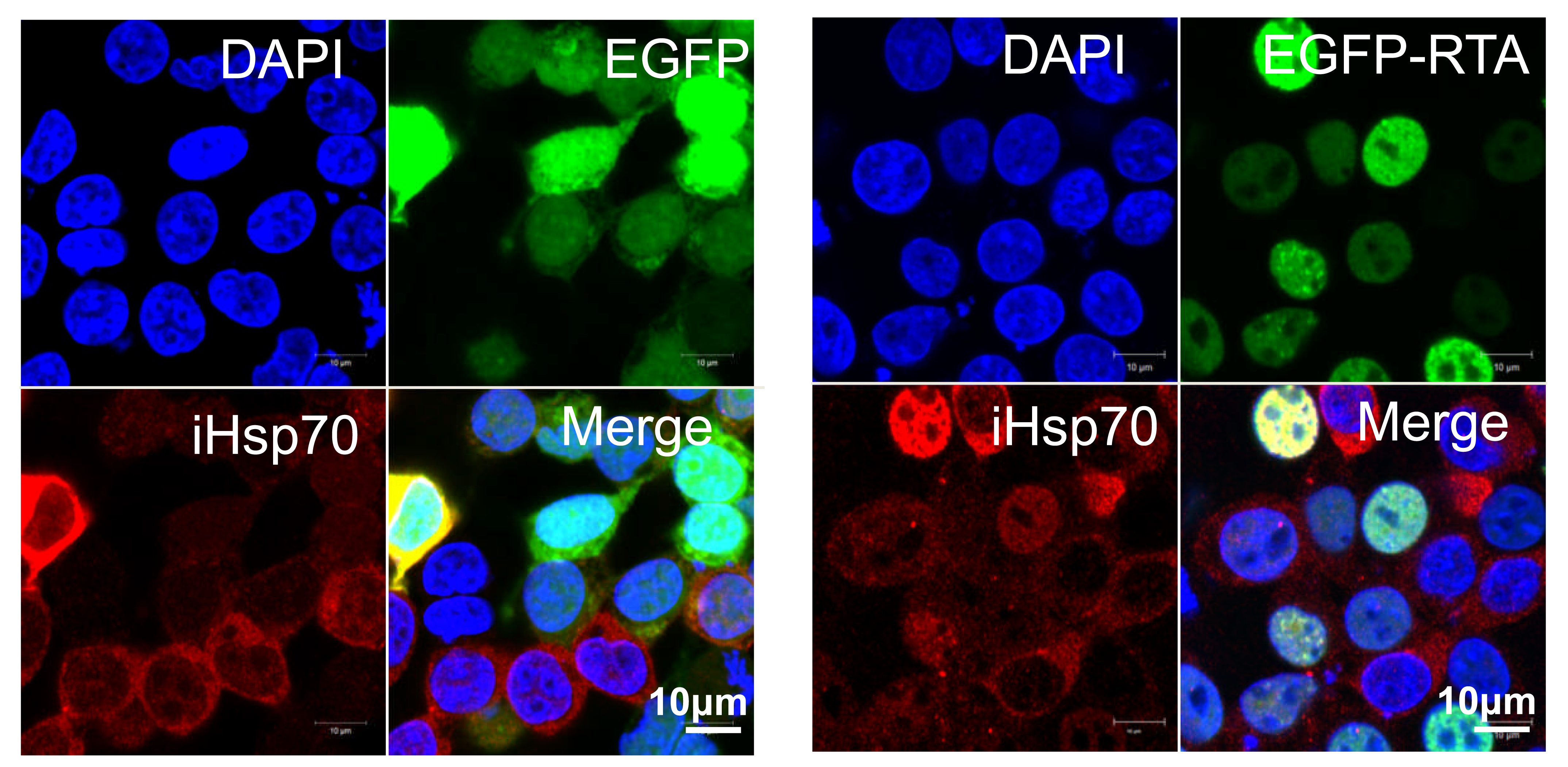

Supplement: S5 Fig — HEK-293T cells were transfected with control pEGFP or pRTA-EGFP for 24 h and then analysed by immunofluorescence. (TIF) [file ppat.1005274.s008.tif]

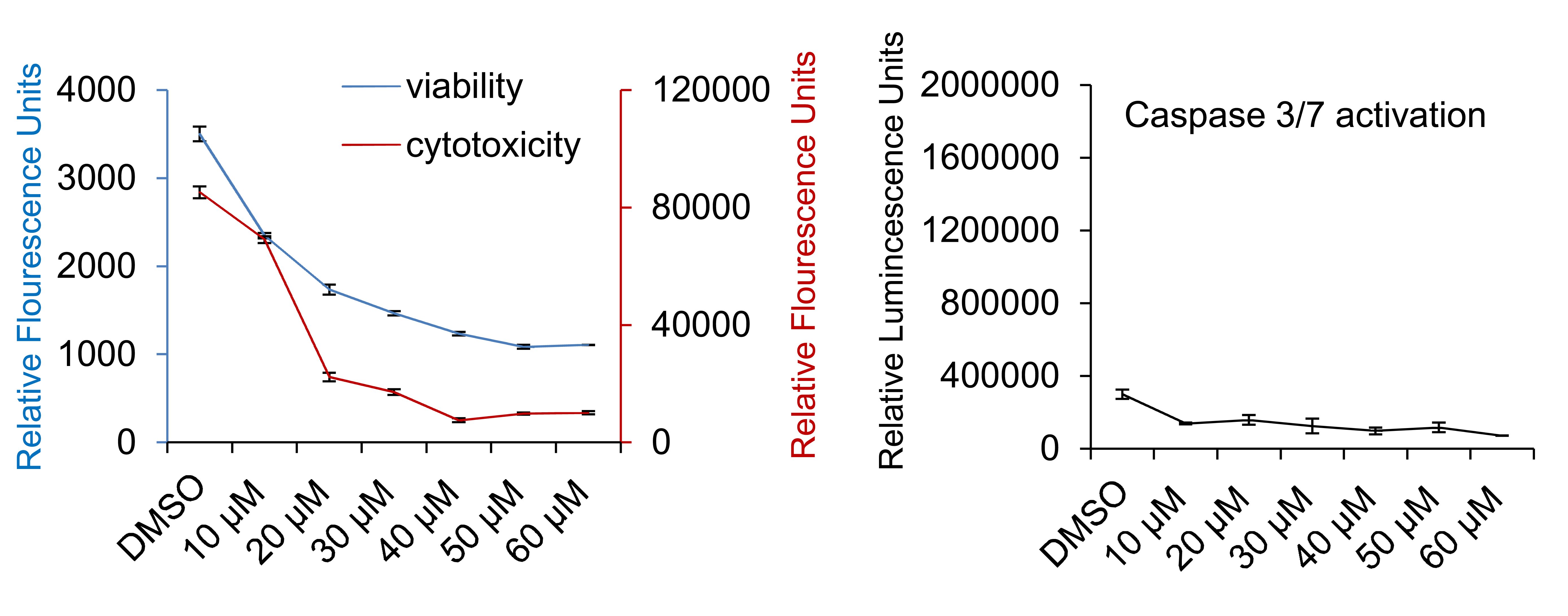

Supplement: S6 Fig — Cytotoxicity of VER-155008 was assessed in cells exposed to increasing inhibitor concentrations for 24 h. Even at 60 μM VER-155008 there was no caspase 3/7 activation compared with DMSO control cells. (TIF) [file ppat.1005274.s009.tif]

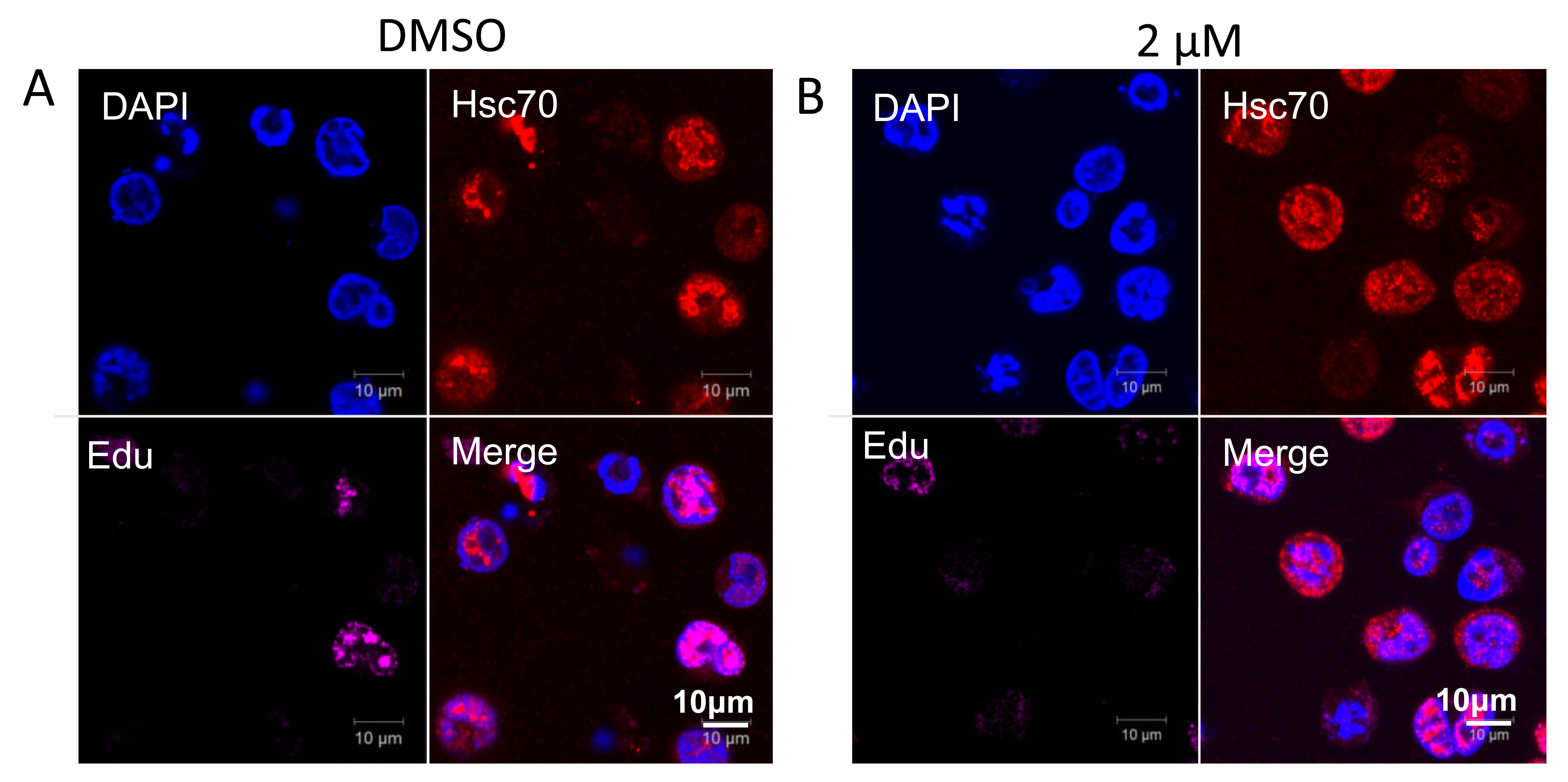

Supplement: S7 Fig — TREx BCBL1-RTA cells were reactivated for 24 h in the presence of control DMSO (0.1%) or 2 μM VER-155008 followed by labelling with Click-iT EdU Alexa Fluor 647 and an antibody specific for Hsc70. (A) In DMSO-treated reactivated cells, Hsc70 formed multiple nuclear foci. Three cells showing viral RTCs filled with viral DNA (Edu-labelled) which co-localised with Hsc70 foci can be seen. (B) Cells treated with VER-155008 displayed Hsc70 protein distributed more equally between the nucleus and cytoplasm and RTCs replicating viral DNA were not as abundant as in DMSO-treated cells. (TIF) [file ppat.1005274.s010.tif]

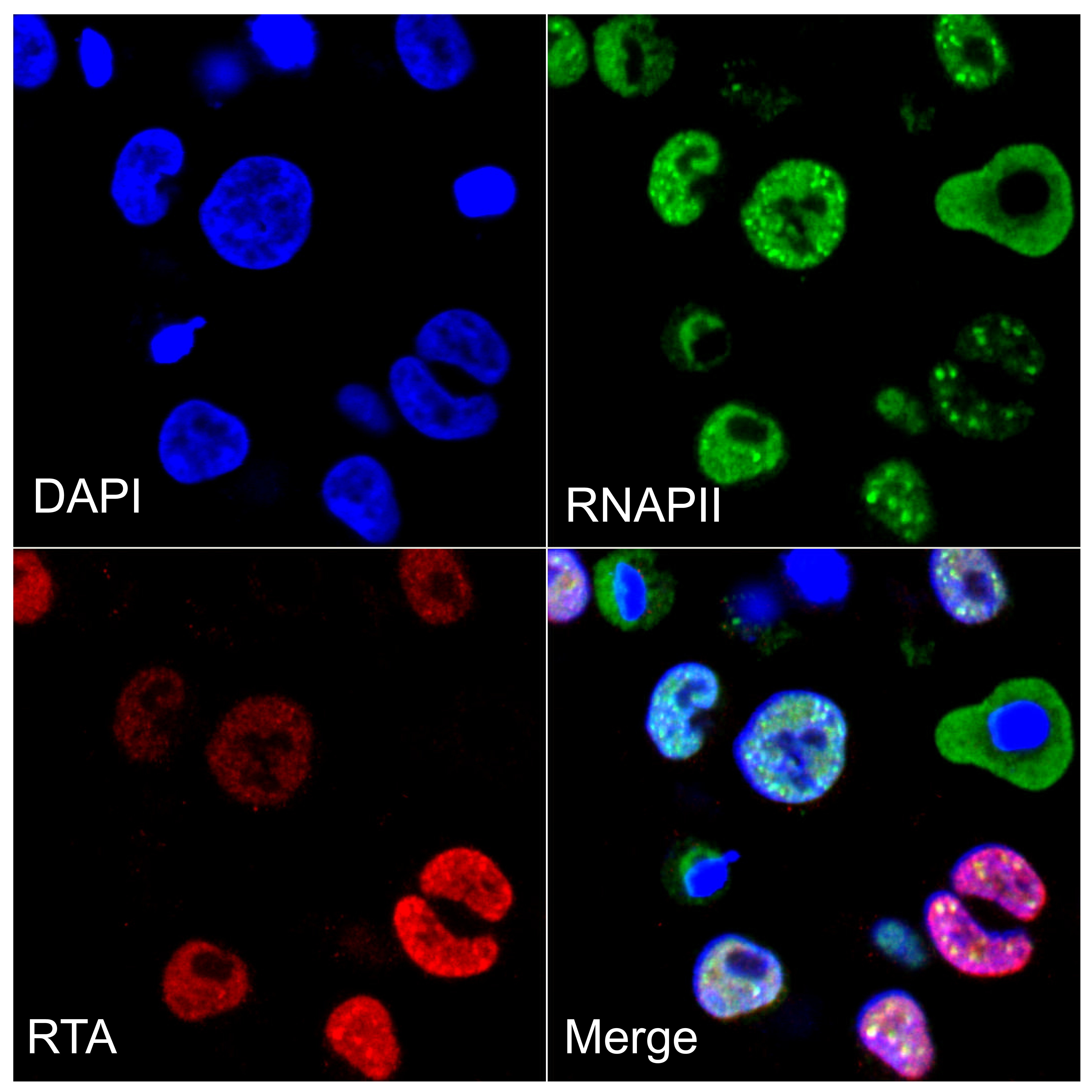

Supplement: S8 Fig — VER-155008 at 2 μM abrogated RNAPII recruitment to KSHV RTCs. (TIF) [file ppat.1005274.s011.tif]

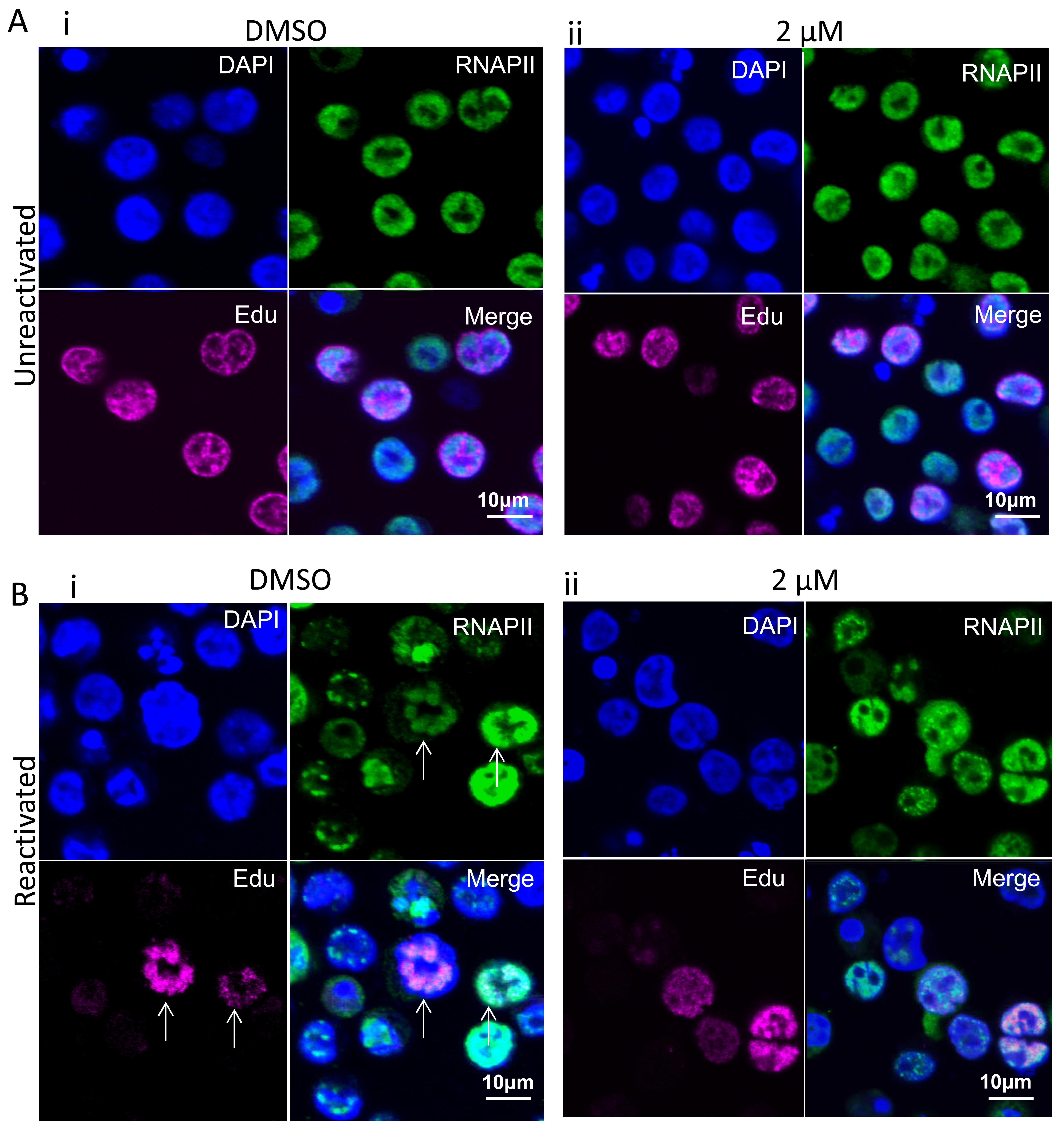

Supplement: S9 Fig — TREx BCBL1-RTA cells remained unreactivated or reactivated for 24 h in the presence of control DMSO (0.1%) or 2 μM VER-155008 followed by labelling with Click-iT EdU Alexa Fluor 647 and an antibody specific for RNAPII (clone CTD4H8). (A) A high proportion of unreactivated TREx BCBL1-RTA cells replicated their cellular DNA (Edu-labelled) in the presence of control DMSO (0.1%) or 2 μM VER-155008. Normal RNAPII localization was observed in these cells, with nuclear RNAPII excluding the nucleoli. (B) In contrast, reactivated cells entered cell cycle arrest as demonstrated by fewer Edu-labelled cells. In the presence of DMSO, multiple RTCs were formed with some replicating viral DNA (white arrows). In cells treated with VER-155008, multiple pre-replicative sites were seen labelled by RNAPII antibody and Edu-labelling was more diffused in the nucleus compared with DMSO-treated cells. (TIF) [file ppat.1005274.s012.tif]
